# Natural selection retains overrepresented out-of-frame stop codons against frameshift peptides in prokaryotes

**DOI:** 10.1186/1471-2164-11-491

**Published:** 2010-09-09

**Authors:** Herman Tse, James J Cai, Hoi-Wah Tsoi, Esther PT Lam, Kwok-Yung Yuen

**Affiliations:** 1 Carol Yu Centre for Infection, Department of Microbiology, The University of Hong Kong, Hong Kong, China; 2 Research Centre of Infection and Immunity, The University of Hong Kong, Hong Kong, China; 3 Department of Biology, Stanford University, Stanford, California, USA; 4 Department of Veterinary Integrative Biosciences, College of Veterinary Medicine, Texas A&M University, Texas, USA

## Abstract

**Background:**

Out-of-frame stop codons (OSCs) occur naturally in coding sequences of all organisms, providing a mechanism of early termination of translation in incorrect reading frame so that the metabolic cost associated with frameshift events can be reduced. Given such a functional significance, we expect statistically overrepresented OSCs in coding sequences as a result of a widespread selection. Accordingly, we examined available prokaryotic genomes to look for evidence of this selection.

**Results:**

The complete genome sequences of 990 prokaryotes were obtained from NCBI GenBank. We found that low G+C content coding sequences contain significantly more OSCs and G+C content at specific codon positions were the principal determinants of OSC usage bias in the different reading frames. To investigate if there is overrepresentation of OSCs, we modeled the trinucleotide and hexanucleotide biases of the coding sequences using Markov models, and calculated the expected OSC frequencies for each organism using a Monte Carlo approach. More than 93% of 342 phylogenetically representative prokaryotic genomes contain excess OSCs. Interestingly the degree of OSC overrepresentation correlates positively with G+C content, which may represent a compensatory mechanism for the negative correlation of OSC frequency with G+C content. We extended the analysis using additional compositional bias models and showed that lower-order bias like codon usage and dipeptide bias could not explain the OSC overrepresentation. The degree of OSC overrepresentation was found to correlate negatively with the optimal growth temperature of the organism after correcting for the G+C% and AT skew of the coding sequence.

**Conclusions:**

The present study uses approaches with statistical rigor to show that OSC overrepresentation is a widespread phenomenon among prokaryotes. Our results support the hypothesis that OSCs carry functional significance and have been selected in the course of genome evolution to act against unintended frameshift occurrences. Some results also hint that OSC overrepresentation being a compensatory mechanism to make up for the decrease in OSCs in high G+C organisms, thus revealing the interplay between two different determinants of OSC frequency.

## Background

The biased codon usage in many genomes is generally believed to result from selection for maximizing translational speed and/or accuracy [1-3], although there is reservation as to what extent the notion can be generalized to all organisms including humans [4,5]. In theory, optimal synonymous codons result in the maximum translational speed. However, the preservation of suboptimal synonymous codons suggests that maximizing translational speed is not the only determinant of codon bias. Synonymous codons may also play a role in gene regulation and generation of the correct protein conformation [6-8]. In some sense translational accuracy may be more important than the speed of translation, and reading frame maintenance is a key functional requirement of translational accuracy as a result of the triplet nature of the genetic code. Given the complexity of the protein synthesis process, it is expected that a certain proportion of all transcriptional and translational processes may go awry even under normal conditions. Additional mechanisms like frameshift suppression and nonsense-mediated mRNA decay help to reduce the incidence and impact of such errors at different steps of the protein synthesis pathway [9,10]. Despite these mechanisms, erroneous proteins cannot be entirely eliminated. Whether and how the cell can deal specifically with these incorrect and often truncated proteins is currently uncertain.

**Figure 1:**
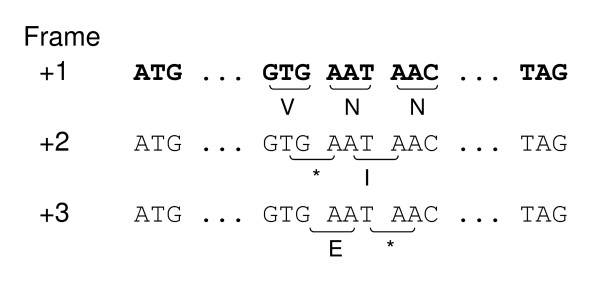
**Illustration showing occurrences of off-frame stop codons (OSCs) in a hypothetical coding sequence**.

The presence of stop codons in the alternate reading frames stop the translation in an incorrect frame (Figure 1) and truncate the portion of frameshift peptides. Although this property of the coding sequence has been known for a long time [11], it has been taken for granted by most researchers. In a study on 72 bacterial genomes, the occurrences of OSCs were examined specifically in detail with the results suggesting that OSC frequency and bias might have influenced the evolution of the bacterial genome [12]. The functional significance of an increased OSC frequency can be explained by the *ambush hypothesis*: OSCs could reduce the metabolic costs of accidental frameshifts, and a positive correlation between the usage of codons and the number of ways codons can be part of hidden stops (i.e., OSCs) is expected [13].

Natural selection can act on the coding sequences to increase OSC frequencies and minimize the influence of translational frameshift errors in different ways. It has been proposed that the genetic code has been optimized to maximize the number of OSCs that could be embedded within the coding sequence [13-15]. On a shorter time-scale, evolution of the coding sequences might contribute by favoring dicodons (codon pairs) that encode OSCs. Presumably it would also be mediated by mechanisms such as synonymous codon usage bias and specific oligonucleotide biases. We noted only one study on single-stranded RNA viral genomes that has directly studied the expected and observed frequencies of these out-of-frame stop codons (OSCs) [16]. Other studies on dicodon bias [17] and overlapping genes [18] have touched upon the topic of OSCs indirectly (that is, the results are not directly relevant to the investigation of relative abundance of OSC, as shown later in the discussion section). A clear limitation is that the calculation of odds ratio adopted by these studies examines only up to two simple types of compositional biases at a time. Furthermore, the reading frame context of coding sequences is frequently ignored, or the individual frames are not taken into account. In general, these limitations led to an unsatisfactory and incomplete description of *k*-mer frequencies that biased the expected number of OSCs, and only the basic association between genomic G+C content and OSCs could be identified [18].

To address the shortcomings of the previous studies, we adopted an approach using Markov models to analyze the coding sequences of prokaryotic genomes. The Markov model is based on the concept of portraying the coding sequence as a Markov chain with defined state path and transition/emission states. This approach is superior in preserving the reading frame context and allowing nested models to account for multiple codon or oligonucleotide biases simultaneously. Additionally it provides the distribution of expected OSC frequencies for each genome and hence allows hypothesis testing in a formal statistical framework. With the availability of nearly a thousand complete prokaryotic genomes at the time of study, we were able to utilize this vast amount of data to look for any significant deviation of OSC frequencies on a per genome basis. We provide evidence in support of the ambush hypothesis and suggest the near-universal presence of selection against translation of frameshift products in prokaryotic genomes.

## Methods

### Genome sequences

Complete prokaryotic genomic sequences were obtained from NCBI GenBank http://www.ncbi.nlm.nih.gov/genomes/lproks.cgi. Protein coding sequences were identified using gene annotations available in the associated GenBank files. Non-protein coding genes (e.g. pseudogenes and rRNA genes), incomplete genes, coding sequences with less than 100 codons, extra-chromosomal sequences and sequences containing ambiguous bases were excluded from analysis. Genomes of bacteria that utilize a non-standard genetic code, such as *Mycoplasma *spp., and mitochondrial genomes were analyzed separately. Additionally, 'artificial metagenomes' were constructed from randomly selected prokaryotic genomes for conducting mixed genome analysis [19].

### Distribution and usage bias of OSCs in alternate reading frames

Absolute OSC counts and OSC densities in the +2 and +3 reading frames (corresponding to the reading frame resultant from +1 and -1 frameshifts respectively) were calculated on a per gene and per genome basis respectively. The absolute OSC counts for the 2 alternate reading frames of all genes in each genome were compared using paired 2-sample t-test. Correction for multiple comparison was done by controlling the false discovery rate at the 5% level using the Benjamini-Hochberg procedure. Relationship of the ratios with G+C content, GC skew and AT skew of protein coding sequences was assessed using multiple regression analysis.

OSC usage bias was defined as the relative codon frequencies of the three stop codons in the two alternate reading frames and assessed by principal component analysis (PCA) as done previously [20]. To investigate the contribution of the G+C content of the 3 codon positions to the OSC usage bias, multiple linear regression analysis was performed with the first principal component as the dependent variable and G+C content of each codon position as regressors. The relative importance of the regressors is estimated using the proportional marginal variance decomposition (PMVD) metric by bootstrapping with 1000 replicates as implemented in the R package 'relaimpo' [21].

### Analysis of OSC relative abundance in the coding sequence

Although the abundance of any trinucleotide can be expressed as a odds ratio given frequencies of its component mononucleotides and dinucleotides (*γ*_xyz _= *f*_xyz _*f*_x _*f*_y _*f*_z _/*f*_xy _*f*_yz _*f*_xnz_) [22], the metric should not be directly applied to the analysis of OSC relative abundance in protein coding sequences because of internal stop codon avoidance in the coding frame. For example, the dicodons TAATTA and TTAATA are equivalent in length, nucleotide and dinucleotide composition, but the former is not allowed in the coding frame while the latter contains an additional OSC. As stop codons in the coding frame could not contribute to formation of OSCs, the number of expected OSC occurrences would also increase. Thus, the avoidance of in-frame stop codons will lead to asymmetry of trinucleotide occurrences in the alternate reading frames.

A Monte Carlo approach is used to estimate the expected OSC frequencies for each genome. To reduce the impact of sampling bias from heavily sampled genera and species such as *Staphylococcus aureus*, we trimmed the original set of available prokaryotic genomes so that on average one genome per genus is included for analysis. Random coding sequences matching the distribution of gene lengths in the target genome were generated using second-order and fifth-order three-periodic Markov models trained on the set of coding sequences, as implemented in the MARKOV package of GenRGenS [23]. The expected frequencies of OSC occurrences in the simulated sequences were then compared to the frequencies observed in the actual sequences using the one-sample t-test, with correction for multiple comparison performed by controlling the false discovery rate at the 5% level using the Benjamini-Hochberg procedure. 200 Monte Carlo trials were performed for each genome.

### Origin of OSC bias in selected genomes

To investigate the origin of an excess of OSC abundance in a genome, we selected several reference genome sequences for further analysis. These included diverse genomes with different degrees of OSC relative abundance. Random coding sequences matching the distribution of gene lengths in the genomes were generated using different codon-based Markov models trained on the sets of coding sequences. These different models accounted for one or more of the following properties of protein coding sequence: codon usage bias, dicodon usage bias and dipeptide bias. As the models were not implemented in the GenRGenS package, the functionality of random sequence generation under these models was implemented in an in-house Perl program. The Mersenne Twister pseudorandom number generation algorithm as implemented in the CPAN module Math::Random::MT::Auto http://search.cpan.org/dist/Math-Random-MT-Auto/ was used, as the numbers generated were known to have suitable statistical properties for Monte Carlo analysis [24]. The OSC frequencies in the alternate frames of the simulated sequences were then compared to that in the actual sequences using the one-sample t-test.

### Relationship between OSC overrepresentation and optimal growth temperature

We explored the possible relationship between genomic OSC overrepresentation and phenotype of the organism. As a test case, we examined the correlation between the degree of OSC overrepresentation and the organism's optimal growth temperature using multiple linear regression analysis, as the growth temperature has been shown to be associated with genomic sequence composition. Regression model comparison was performed using ANOVA and stepwise variable selection using Akaike information criterion. Data on optimal growth temperature of the organisms were obtained from previous studies [25,26].

### Statistical analysis

Statistical analysis was performed using R version 2.10.1. All p-values reported are for a two-tailed test, and p < 0.05 is considered statistically significant.

## Results

### Distribution of OSCs in alternate reading frames

**Figure 2:**
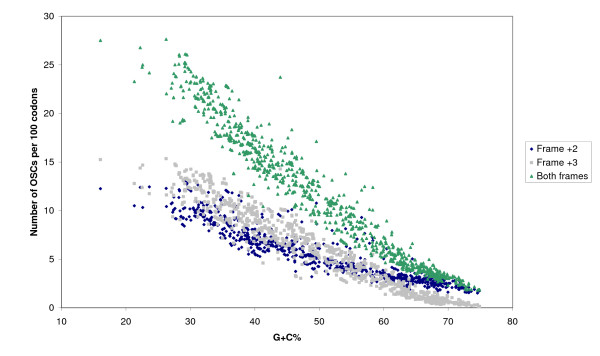
**Plot of total and per-frame OSC frequencies against G+C content of coding sequences, showing a roughly linear decrease in OSC frequencies as the G+C content increase**.

Among the 990 analyzed genomes (Additional File 1, Supplementary Table S1), the mean OSC densities range from 1.82 to 27.6 per 100 codons. When the OSCs are analyzed by reading frames, the mean OSC densities for frame +2 range from 1.52 to 13.2 per 100 codons and the mean OSC densities for frame +3 range from 0.11 to 15.3 per 100 codons (Figure 2). OSC densities for both reading frames decrease with increasing G+C content of the coding region, and the rate of decrease was greater for frame +3. The mean OSC density was found to be very strongly and negatively correlated with the G+C content in the coding regions by linear regression (p < 0.0001; r = -0.9721, R^2 ^= 0.9451). The negative correlation is consistent with our expectations as all three stop codons are AT-rich. Multiple linear regression analysis showed that accounting for GC skew of coding regions and genome length slightly improved the model fit (p < 0.0001; adjusted R^2 ^= 0.9608) when compared to the original model by ANOVA (p < 0.0001).

**Figure 3:**
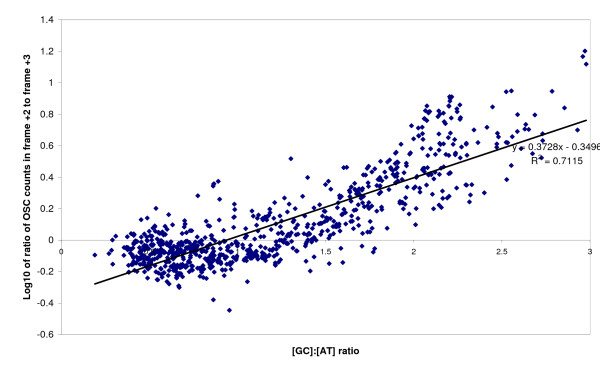
**Plot of GC ratio ([GC]:[AT] ratio) of coding sequences against OSC frame bias expressed in log_10 _of the ratio of OSC counts in frame +2 to frame +3**.

When comparing absolute OSC counts on a per gene basis, 421 out of 990 genomes (42.5%) have a significantly higher OSC count in the +2 reading frame, while 520 (52.5%) have a higher OSC count in the +3 reading frame. The ratio of absolute OSC counts in the 2 alternate reading frames on a per gene basis was found to be correlated with the GC ratio (ratio of [GC]:[AT] content) of coding sequences (Figure 3). The x-intercept of the regression line is noted to be 0.9378. Hence, OSC occurrences for frames +2 and +3 are predicted to be approximately equal at a G+C content of 48.40%, as exemplified by the case of *Dehalococcoides *sp. VS with a G+C content of 48.15%. Multiple regression analysis showed that accounting for coding genome size, GC skew and codon positional effects improved the model fit (adjusted R^2 ^= 0.7530) when compared to the original model by ANOVA (p < 0.0001), although the G+C content at codon position 1 was found not to contribute significantly to the regression model.

### OSC usage bias in alternate reading frames

**Figure 4:**
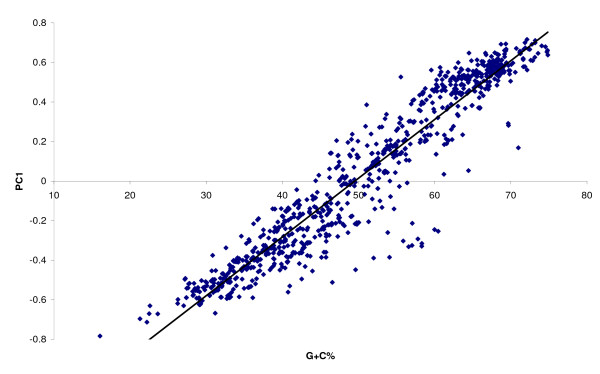
**Plot of the first PCA axis against G+C content of coding sequences, showing a strong linear correlation and suggesting that G+C% is the principal determinant of overall OSC usage bias**.

The first three axes from PCA of OSC usage bias accounted for 92.7%, 3.9% and 2.5% of the total variance respectively (Additional File 1, Supplementary Figure S1). Linear regression of the first PCA axis with G+C content showed a strong correlation (R^2 ^= 0.9151; p < 0.0001) (Figure 4). Among the OSCs, the TAG frequency in frame +3 was the only one not to be associated with the first PCA axis at all (Additional File 1, Supplementary Figure S2), and hence not considered to be associated with the G+C content of the coding sequence.

**Figure 5:**
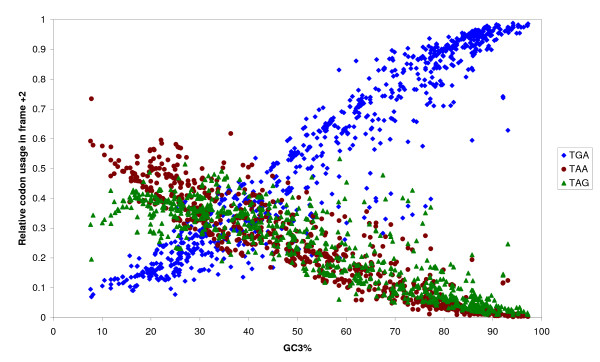
**Plot of relative OSC usage in frame +2, showing TGA usage increases with G+C content of coding sequence while TAA and TAG usage decrease**.

**Figure 6:**
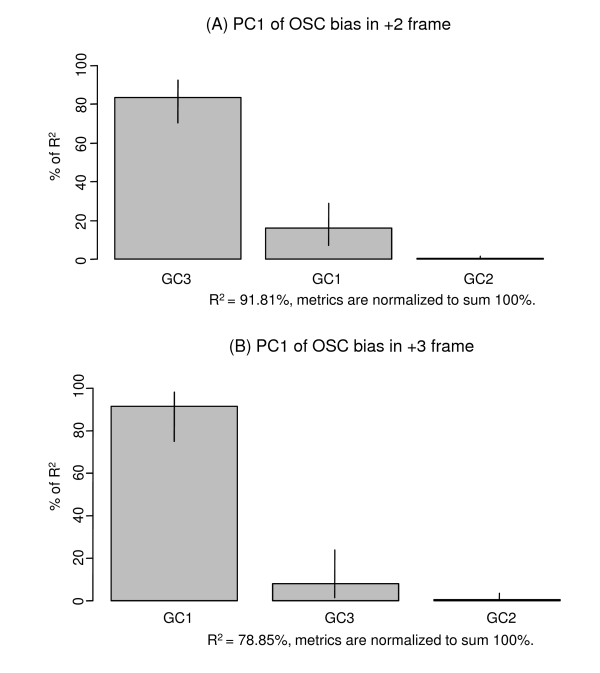
**Plot of relative importance of G+C content in different codon positions in its correlation with OSC usage bias in the +2 frame (A) and the +3 frame (B)**. Note that the proportion of total variance explained, R^2^, is much smaller for the +3 frame (78.75%) than the +2 frame (91.81%), which is consistent with the PCA results shown in Additional File 1, Supplementary Figure 2.

The OSC usage bias in each alternate reading frame is considered below individually. For the +2 frame, OSC occurs in the dicodon sequence N**T**[**GA**|**AA**|**AG**]NN in the protein coding frame. As the third codon position is the most variable codon position due to degeneracy of the genetic code, variation of G+C content at the third codon position (GC3) should have the greatest effect on OSC usage bias for frame +2. As expected, relative frequencies of TAA and TAG for frame +2 decreased with increasing GC3 while that of TGA increased (Figure 5), despite the equivalent nucleotide composition of TAG and TGA. However, as G+C content in all codon positions are highly correlated, the OSC usage bias would also be highly correlated with G+C content at other codon positions. To resolve the situation, the relative contribution of the G+C content at different codon positions to OSC usage bias was estimated by the PMVD metric, which emphasizes the conditional importance of one regressor in the presence of other regressors [27] and hence can account for the significant multicollinearity among the G+C content at different codon positions. Results showed that GC3 is the dominant independent regressor of OSC usage bias in the +2 frame (Figure 6A), suggesting that correlation of GC1 or GC2 with OSC usage bias is mostly a result of their multicollinearity with GC3.

The coding sequence prerequisite for OSC occurrence in the +3 frame is the dicodon NN**T**[**GA**|**AA**|**AG**]N. In this case, OSC usage bias is not directly affected by GC3 (which should only affect overall OSC frequency) but by the first and second codon positions of the second half of the dicodon. Regression analysis confirmed that GC1 is the dominant independent regressor (Figure 6B).

### Analysis of OSC relative abundance in the coding sequence

Almost all the examined genomes (334/342; 97.7%) showed overrepresentation of OSCs in the +2 frame when compared to frequencies predicted by the second-order three-periodic Markov model. This model accounted for the codon position-specific trinucleotide bias including codon usage bias. Only 3 genomes (0.9%) showed a statistically significant underrepresentation of OSCs in the +2 frame under the same model. When compared to the OSC frequencies predicted by the fifth-order three-periodic Markov model, 185 genomes (54.1%) still showed statistically significant overrepresentation of OSCs in the +2 frame, while 26 genomes (7.6%) showed underrepresentation of OSCs in the same frame. For the +3 frame, 57 and 53 genomes (16.7% and 15.5%) showed overrepresentation and underrepresentation of OSCs respectively when compared to frequencies predicted by the second-order three-periodic Markov model. When examined under the fifth-order three-periodic Markov model, the number of genomes with OSC overrepresentation in the +3 frame greatly increased to 306 (89.5%), while only 4 (1.2%) showed underrepresentation.

**Figure 7:**
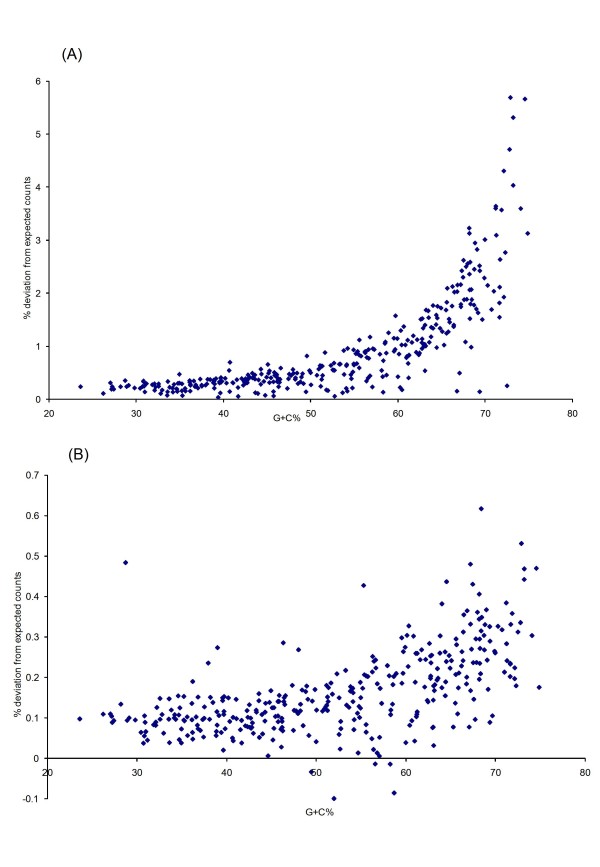
**Plots of percentage deviation of observed OSC counts from expected counts under (A) second-order and (B) fifth-order three-periodic Markov models against G+C content of coding regions**. A positive percentage deviation signifies overrepresentation of OSCs.

When both alternate reading frames were considered together, 339 genomes (99.1%) showed OSC overrepresentation under the second-order three-periodic Markov model, while 319 genomes (93.3%) showed OSC overrepresentation under the fifth-order three-periodic Markov model. The percentage deviations of the observed from mean expected OSCs were found to range from -0.0343% to +5.69% and -0.111% to +0.616% under the second- and fifth-order three-periodic Markov models respectively. There are significant positive correlations between G+C content and the degrees of OSC overrepresentation under both models (Spearman's rank correlation coefficient = 0.838 and 0.630 for the second- and fifth-order three-periodic Markov models respectively) (Figures 7A and 7B).

Mixed genome analysis was conducted with 8 artificial metagenomes with sizes ranging from 5.2 to 12.2 MB. OSC overrepresentation was found in all cases, ranging from 0.084 to 0.841%, under both the second- and fifth-order three-periodic Markov models. These results indirectly suggested that the phenomenon of OSC overrepresentation is stable to distant horizontal gene transfer, and should apply to presently uncharacterized genomes that may have arisen from extensive horizontal gene transfer with significant sequence compositional diversity and phylogenetic incongruence [28,29].

### Origin of OSC bias in selected genomes

The expected frequencies of OSC occurrences in the selected genomes under the different models were shown in Table 1. The results showed that expected OSC frequencies calculated using codon-based models showed significantly greater deviation from observed values when compared to the three-periodic nucleotide-based models.

**Table 1:** Expected versus observed OSC frequencies of selected genomes under models of different compositional biases.

Organism	Phylogenetic division	G+C% of coding region	Expected OSC frequency under different compositional models					Observed OSC frequency
				
			Codon usage bias	Dipeptide bias	Dicodon bias	**Trinucleotide bias (2**^**nd **^**order Markov model)**	**Hexanucleotide bias (5**^**th **^**order Markov model)**	
*Laribacter hongkongensis*	Beta-proteobacteria	63.5%	6.068 ± 0.025	6.190 ± 0.024	5.024 ± 0.022	4.947 ± 0.021	5.020 ± 0.021	5.030

*Staphylococcus aureus*	Firmicutes	33.6%	19.789 ± 0.038	20.158 ± 0.037	20.501 ± 0.042	20.483 ± 0.044	20.482 ± 0.042	20.512

*Yersinia enterocolitica*	Gamma-proteobacteria	48.4%	12.530 ± 0.026	12.825 ± 0.028	12.902 ± 0.026	12.848 ± 0.028	12.890 ± 0.030	12.912

*Thermotoga maritima*	Thermotogae	46.4%	12.626 ± 0.042	12.177 ± 0.039	10.532 ± 0.042	10.501 ± 0.038	10.519 ± 0.037	10.534

*Deinococcus radiodurans*	Deinococcus-Thermus	67.7%	4.619 ± 0.021	5.118 ± 0.023	4.371 ± 0.021	4.295 ± 0.022	4.369 ± 0.021	4.375

*Pyrococcus furiosus*	Euryarchaeota	41.1%	17.385 ± 0.046	16.927 ± 0.044	16.567 ± 0.050	17.520 ± 0.048	17.557 ± 0.046	17.574

Values are expressed as (mean ± SD) number of OSC per 100 codons.

All the simpler Markov models chosen were nested within the more complex models (Additional File 1, Supplementary Figure S3), with the codon usage bias model being the simplest and the fifth-order three-periodic Markov model being the most complex. Hence, comparisons could be made between any two nested models to infer how different oligonucleotide or codon biases contributed to the predicted OSC frequency as shown in Table 1.

### OSC selection in genomes utilizing alternate genetic codes

Some prokaryotes, such as the *Entomoplasmatales *and *Mycoplasmatales*, utilize an alternate genetic code in which UGA is not a stop codon [30]. Other genetic codes with non-standard stop codons are also utilized in mitochondrial genomes. We extended the analysis under the second-order three-periodic Markov model to these genomes with alternate genetic codes (Table 2) which showed varying results. There was clear evidence for OSC overrepresentation in the genomes of *Mesoplasma florum *and *Mycoplasma agalactiae*, while OSC underrepresentation is present in the genome of *Ureaplasma urealyticum*. For the mitochondrial genomes, the results are similar with a mix of OSC underrepresentation and overrepresentation among the various species. However, their small sizes and the small number of genes limited the statistical significance of the results, which illustrated that the present method is less useful in the analysis of organeller and viral genomes.

**Table 2:** Expected versus observed OSC frequencies of selected genomes with non-standard genetic codes.

Species	Expected OSC counts (per 100 codons) (mean ± SD)	Observed OSC counts (per 100 codons)	p-value
*Entomoplasmatales *&*Mycoplasmatales *(translation table 4)			

*Mesoplasma florum *L1	16.653 ± 0.066	17.233	1.81 × 10^-190^

*Mycoplasma agalactiae *PG2	16.698 ± 0.069	17.053	4.06 × 10^-145^

*Ureaplasma urealyticum*	18.169 ± 0.076	18.154	0.00512

Vertebrate mitochondria (translation table 2)			

*Homo sapiens*	16.107 ± 0.688	16.176	0.162

*Rattus norvegicus *strain Wistar	17.937 ± 0.674	17.950	0.777

Yeast mitochondria (translation table 3)			

*Saccharomyces cerevisiae*	23.178 ± 0.399	23.154	0.408

*Candida glabrata*	25.924 ± 0.675	26.008	0.0809

Ascidian mitochondria (translation table 13)			

*Ciona savignyi*	18.447 ± 0.545	18.506	0.127

Values are expressed as number of OSC per 100 codons.

### Relationship between OSC overrepresentation and optimal growth temperature

Prokaryotes included in our analysis were classified into one of the following 4 categories: psychrophiles, mesophiles, thermophiles and hyperthermophiles. The degree of OSC overrepresentation was found to correlate negatively with the optimal growth temperature of the organism after correcting for the G+C% and AT skew of the coding sequence (p = 3.97 × 10^-5^). The relationship between OSC overrepresentation and optimal growth temperature was also supported by stepwise variable selection on the multiple linear regression model using Akaike information criterion.

## Discussion

Ever since the recognition of the reading frame in ribosomal translation of protein coding sequence, it has been realized that off-frame stop codons play a role in avoiding production of erroneous protein products. At the very least, erroneous peptides resulted from frameshift have reduced function or be entirely non-functional, and consume precious cellular resources; and in the worst case, they may be toxic and interfere with normal cellular metabolism. Hence, it is natural to postulate that OSCs would be selected for in the course of genome evolution. An increase in the occurrences of OSCs results in more truncations of the erroneous peptides due to frameshifts, and leads to less metabolic wastage and potentially less toxic products. In agreement with this line of reasoning, there is empirical evidence that protein production increases with the number of OSCs in the coding gene [31].

Our study is divided into two main parts. Firstly, we showed that GC bias in the coding sequences is the primary determinant of OSC frequencies, consistent with the results of a smaller study [12]. Furthermore, with a lone exception, individual OSC biases are also primarily determined by the G+C content of the coding sequences. Hence, these results establish the need to account for the effect of nucleotide compositional bias on OSC frequencies. In the second part of the study, we investigated the effects of higher order compositional biases, like dinucleotide, hexanucleotide and dicodon biases, that have been recognized in genomes previously [32-35]. Markov modeling provides a straightforward and natural way of describing these biases and hence allow for the estimation of the effects of the different biases on OSC frequencies. Perhaps the biggest advantage of Markov modeling in the context of this study is the ease with which nested models could be developed and compared. These models would have been more complicated to implement using previous approaches like *k*-mer shuffling [36] or odds ratio of word counts [22]. The generation of random genomes under different models greatly facilitates the study of a wide range of genomic features in relation to the underlying compositional biases, and the flexibility of the approach is only limited by the computational expense of the associated Monte Carlo method.

The selection of Markov models examined represents a balance of biological relevance and statistical considerations. Markov models with orders of six or above were not examined in the present analysis due to the limited size of prokaryotic genomes resulting in insufficient sample sizes for parameter estimation. Furthermore, except for special cases like palindromic sequences, it is uncertain whether any biological mechanism exists to produce such a high-order oligonucleotide bias. The same argument applies to the codon-based Markov models. At the other end of the spectrum, Markov models simpler than second-order nucleotide-based models reflect only simple nucleotide composition or dinucleotide bias and could not account for the absence of in-frame stop codons. The choice of second- and fifth-order nucleotide-based three-periodic Markov models as used in the present study is not arbitrary. Previous work in applying Markov models to gene prediction have shown them to be the most useful models for describing protein coding sequences [37,38], and are important in the majority of current gene prediction programs.

The results of the present study supported the general presence of selection for OSC in prokaryotic genomes, with more than 93% of examined genomes clearly showing OSC overrepresentation under the nucleotide-based Markov models. In further support for the ambush hypothesis, the magnitude of OSC overrepresentation is found to be significantly correlated with G+C content. The results showed that genomes with higher G+C content tend to have a higher degree of OSC overrepresentation. As the same genomes have less OSCs as shown in the first part of the study, the increased OSC overrepresentation might well be a compensatory mechanism to boost the number of OSCs. This observation highlighted a previously overlooked aspect of the ambush hypothesis -- the selection for OSCs can occur simultaneously at multiple levels and there exists a complex layer of interaction among them.

On the other hand, the magnitude of OSC overrepresentation was found to be quite modest, and does not exceed 6% in the most pronounced case. However, before dismissing the practical significance of the effect, it should be reminded that the present calculations were done on a per genome basis. Taking the case of *Yersinia enterocolitica *as an example, OSC overrepresentation of around 0.64 per 1000 codons in its 4.6 MB genome would translate to an excess of over 800 OSCs. Even a weak selection of OSCs can sometimes produce unexpected and significant effects in the phenotype, as exemplified by the recent discovery of a positive association between numbers of mitochondrial OSCs and the accuracy of vertebrate morphogenetic development [39]. In our results, OSC overrepresentation is negatively correlated with optimal growth temperature of the organism in general. We hypothesize that low temperatures may promote non-specific binding of transcriptional or initiation factors to incorrect sites and thus confer a selectional advantage to a greater abundance of OSCs. While there is insufficient data to indicate that translational or transcriptional error rates are elevated in low temperatures, we note that our proposed mechanism shares conceptual and functional similarities with the arrest of initiation factor-dependent translation initiation mediated by the cold shock response [40].

Our results provide a picture of OSC selection averaged over the whole genome. As the probability and adverse effects associated with frameshift occurrences may vary with individual genes, so will the "selection pressure" to incorporate additional OSCs into its coding sequence. Thus, it is possible that excess OSCs are not evenly distributed but more concentrated in a subset of genes, in which they may exert a pronounced effect against frameshift peptide translation. Logically, genes with frameshift-prone slippage regions such as homopolymeric tracts [41] would benefit most from excess OSCs. Alternatively, it may be possible that highly expressed genes would also be under selection for more OSCs, as the absolute number of errors would increase with greater transcription and translation activity. While the uneven distribution of OSCs in the genes and genomes was not explored in the present study, we calculated the ratio of OSCs in the +2 and +3 frames, which showed significant variation among the different genomes and could not be fully explained by the genomic G+C content as shown in figure 2. With respect to the importance of the physical distribution of OSCs, the concept of the "tri-frame model" and its application of the ribosome occupancy distribution may provide a useful framework for understanding the uneven distribution of OSCs with respect to reducing mistranslation and modulating gene expression [42].

The diversity of results from the detailed analysis on selected genomes is useful in showing that codon or dipeptide biases alone could not explain the near-universal observation of OSC overrepresentation in prokaryotic genomes. We notice that the expected OSC frequencies under the dicodon bias model closely match the actually observed freqencies, suggesting that dicodon bias may play an important part in affecting OSC occurrences. However, there appears to be exceptions, like *Pyrococcus furiosus*, for which the dicodon bias model failed to model the observed OSC frequency (Table 1). A related observation is that the simpler models appear inadequate in describing the compositional biases in the coding sequences. For example, the zeroth-order codon-based Markov model assumed complete independence of each codon from its neighbors, thus implying the absence of dinucleotide or other compositional biases across codon boundaries. Hence, the presence of biologically inaccurate assumptions renders the model irrelevant for comparison. Since the above results have largely ruled out the role of the lower-order compositional biases, another prime candidate for contributing to the OSC overrepresentation is local synonymous codon usage. This possibility could not be confirmed with the current methods and deserve exploration in future studies.

Maintenance of the reading frame of a coding sequence is a complex and error-prone process. During the transfer of genetic information from DNA to protein, errors resulting in frameshifts may occur during DNA replication, mRNA transcription and ribosomal translation. It is also possible that some errors may arise from DNA and RNA mutations, that may occur spontaneously or be induced by mutagens. To minimize the metabolic impact of these errors, the cell has several layers of defense. Firstly, the relevant cellular processes have been highly optimized to avoid the errors in the first place. For example, higher fidelity of DNA replication could be achieved with the use of proofreading DNA polymerase. Next, if errors had nonetheless occurred, the appropriate response mechanisms will be engaged. Damaged DNA may be recognized and corrected with the cellular DNA repair machinery while translational frameshifts may be reduced with frameshift suppressor tRNAs. Finally, the cell possesses a certain degree of metabolic robustness to resist the negative effects of these errors, such as the presence of alternative metabolic pathways. In this framework, the selection of OSCs in coding sequences could be considered a passive second layer of defense against frameshift errors. It is uncertain if there is greater selective pressure against transcriptional than translational frameshifts, given that the effect of OSCs is identical in both cases. A related mechanism identified to play a similar role in potentially reducing mistranslation errors is the selection on codon-pair context during gene evolution to maximize mRNA decoding fidelity by optimizing translational efficiency [43]. This effect would be independent of and additive to that provided by OSCs.

As a final note, we would like to explore the differences between the present results and those from a previous study [17]. In that study, the authors examined the preferred and avoided dicodons in different genomes and noted that some avoided dicodons allows for out-of-frame UAA/UAG stop codons (but not UGA stop codons) in alternate reading frames. However, to put their findings in perspective, we noticed that the set of preferred dicodons also included dicodons that encode such OSCs, and no calculations were performed to confirm whether the net effect of the dicodon bias actually decreased OSC frequencies. Thus, there was no direct demonstration of OSC avoidance in the genomes. More importantly, by calculating the odds ratio of dicodon frequencies based on the constituent codon frequencies, they have shown only the effect of dicodon bias on overall OSC frequencies and not the actual difference between observed and expected OSC frequencies. For instance, our analysis on *Laribacter hongkongensis *strain HLHK9 [44] (Table 1) revealed OSC overrepresentation in its genome though its dicodon bias actually decreased the OSC abundance relative to its codon usage and dipeptide biases. Hence, it is clear that the results from the previous study are not sufficiently informative in the investigation of OSC selection in genomes.

## Conclusions

We have presented the largest and most comprehensive study to date of OSCs in prokaryotic genomes using Markov models and the Monte Carlo method. Results showed widespread overrepresentation of OSCs and the degree of overrepresentation increases with G+C content of the coding sequence. The latter observation is postulated to be a compensatory mechanism to make up for the decrease of OSC frequency with G+C content. Taken together, the findings of the study provided evidence in support of selection for OSCs at the genomic level, in agreement with the ambush hypothesis which stated that OSCs can reduce the metabolic cost associated with unintended frameshift events.

## Authors' contributions

HT conceived and designed the study. HT and JJC wrote the manuscript. HT, HWT and EPTL performed data analysis. KYY performed critical revision of the manuscript for important intellectual content. All authors have read and approved the manuscript.

## Supplementary Material

Additional file 1 **Supplementary Table S1 and Supplementary Figures S1 to S3**. Table S1, list of analyzed genomes; Figure S1, scree plot of the principal component analysis (PCA) on the relative codon frequencies of off-frame stop codons (OSC); Figure S2, biplot of the PCA results; Figure S3, relationship of the Markov models used. Click here for file

Additional file 2 **Results from Monte Carlo simulations of OSC frequencies in prokaryotic genomes**. Excel file containing simulated OSC frequencies under different Markov models from each Monte Carlo run. Click here for file
