# Supplementary material for: Natural selection retains overrepresented out-of-frame stop codons against frameshift peptides in prokaryotes
Source: BMC Genomics. 2010 Sep 9;11:491. doi: 10.1186/1471-2164-11-491 (PMC2996987; doi:10.1186/1471-2164-11-491)

Supplementary Table S1. List of the complete genomes included in the analysis.

GenBank accession no.	Organism name
NC_009925	[*]*Acaryochloris marina* MBIC11017
NC_013209	[*]*Acetobacter pasteurianus* IFO 3283-01
NC_010163	[*]*Acholeplasma laidlawii* PG-8A
NC_013124	[*]*Acidimicrobium ferrooxidans* DSM 10331
NC_009484	*Acidiphilium cryptum* JF-5
NC_011761	[*]*Acidithiobacillus ferrooxidans* ATCC 23270
NC_011206	*Acidithiobacillus ferrooxidans* ATCC 53993
NC_012483	[*]*Acidobacterium capsulatum* ATCC 51196
NC_008578	[*]*Acidothermus cellulolyticus* 11B
NC_008752	[*]*Acidovorax avenaes* subsp. *citrulli* AAC00-1
NC_008782	*Acidovorax* sp. JS42
NC_011586	[*]*Acinetobacter baumannii* AB0057
NC_011595	*Acinetobacter baumannii* AB307-0294
NC_010611	*Acinetobacter baumannii* ACICU
NC_009085	*Acinetobacter baumannii* ATCC 17978
NC_010410	*Acinetobacter baumannii* AYE
NC_010400	*Acinetobacter baumannii* SDF
NC_005966	*Acinetobacter* sp. ADP1
NC_009053	[*]*Actinobacillus pleuropneumoniae* L20
NC_010278	*Actinobacillus pleuropneumoniae* serovar 3 str. JL03
NC_010939	*Actinobacillus pleuropneumoniae* serovar 7 str. AP76
NC_009655	*Actinobacillus succinogenes* 130Z
NC_013093	[*]*Actinosynnema mirum* DSM 43827
NC_008570	[*]*Aeromonas hydrophila* subsp. *Hydrophila* ATCC 7966
NC_009348	*Aeromonas salmonicida* subsp. *salmonicida* A449
NC_000854	[*]*Aeropyrum pernix* K1
NC_013416	[*]*Aggregatibacter actinomycetemcomitans* D11S-1
NC_012913	*Aggregatibacter aphrophilus* NJ8700
NC_011985 (chromosome 1); NC_011983 (chromosome 2)	[*]*Agrobacterium radiobacter* K84
NC_003062	*Agrobacterium tumefaciens* str. C58
NC_011989 (chromosome 1); NC_011988 (chromosome 2)	*Agrobacterium vitis* S4
NC_010655	[*]*Akkermansia muciniphila* ATCC BAA-835
NC_008260	[*]*Alcanivorax borkumensis* SK2
NC_013205	[*]*Alicyclobacillus acidocaldarius* subsp. *acidocaldarius* DSM 446
NC_011312 (chromosome 1); NC_011313 (chromosome 2)	[*]*Aliivibrio salmonicida* LFI1238
NC_008340	[*]*Alkalilimnicola ehrlichei* MLHE-1
NC_009633	[*]*Alkaliphilus metalliredigens* QYMF
NC_009922	*Alkaliphilus oremlandii* OhILAs
NC_011138	[*]*Alteromonas macleodii* ‘Deep ecotype’

NC_013385	[*]*Ammonifex degensii* KC4
NC_007413	[*]*Anabaena variabilis* ATCC 29413
NC_012034	[*]*Anaerocellum thermophilum* DSM 6725
NC_013171	[*]*Anaerococcus prevotii* DSM 20548
NC_007760	[*]*Anaeromyxobacter dehalogenans* 2CP-C
NC_011891	*Anaeromyxobacter dehalogenans* 2CP-1
NC_009675	*Anaeromyxobacter* sp. Fw109-5
NC_011145	*Anaeromyxobacter* sp. K
NC_013532	[*]*Anaplasma centrale* str. Israel
NC_012026	*Anaplasma marginale* str. Florida
NC_004842	*Anaplasma marginale* str. St Maries
NC_007797	*Anaplasma phagocytophilum* HZ
NC_011567	[*]*Anoxybacillus flavithermus* WK1
NC_000918	[*]*Aquifex aeolicus* VF5
NC_000917	[*]*Archaeoglobus fulgidus* DSM 4304
NC_009850	[*]*Arcobacter butzleri* RM4018
NC_006513	[*]*Aromatoleum aromaticum* EbN1
NC_008711	[*]*Arthrobacter aurescens* TC1
NC_011886	*Arthrobacter chlorophenolicus* A6
NC_008541	*Arthrobacter* sp. FB24
NC_007716	[*]Aster yellows witches'-broom phytoplasma AYWB
NC_013203	[*]*Atopobium parvulum* DSM 20469
NC_008702	[*]*Azoarcus* sp. BH72
NC_009937	*Azorhizobium caulinodans* ORS 571
NC_012560	*Azotobacter vinelandii* DJ
NC_009725	[*]*Bacillus amyloliquefaciens* FZB42
NC_012659	*Bacillus anthracis* str. A0248
NC_003997	[*]*Bacillus anthracis* str. Ames
NC_007530	*Bacillus anthracis* str. ‘Ames Ancestor’
NC_012581	*Bacillus anthracis* str. CDC 684
NC_005945	*Bacillus anthracis* str. Sterne
NC_012472	*Bacillus cereus* 03BB102
NC_011658	*Bacillus cereus* AH187
NC_011773	*Bacillus cereus* AH820
NC_004722	*Bacillus cereus* ATCC 14579
NC_003909	*Bacillus cereus* ATCC 10987
NC_011725	*Bacillus cereus* B4264
NC_009674	*Bacillus cereus* subsp. *cytotoxis* NVH 391-98
NC_011772	*Bacillus cereus* G9842
NC_011969	*Bacillus cereus* Q1
NC_006274	*Bacillus cereus* E33L
NC_006582	*Bacillus clausii* KSM-K16
NC_002570	*Bacillus halodurans* C-125
NC_006270	*Bacillus licheniformis* ATCC 14580 (UG)
NC_006322	*Bacillus licheniformis* ATCC 14580 DSM 13 (NB)
NC_009848	*Bacillus pumilus* SAFR-032
NC_000964	*Bacillus subtilis* subsp. *subtilis* str. 168
NC_008600	*Bacillus thuringiensis* str. Al Hakam

NC_005957	*Bacillus thuringiensis* serovar *konkukian* str. 97-27
NC_010184	*Bacillus weihenstephanensis* KBAB4
NC_003228	[*]*Bacteroides fragilis* NCTC 9434
NC_006347	*Bacteroides fragilis* YCH46
NC_004663	*Bacteroides thetaiotaomicron* VPI-5482
NC_009614	*Bacteroides vulgatus* ATCC 8482
NC_008783	[*]*Bartonella bacilliformis* KC583
NC_012846	*Bartonella grahamii* as4aup
NC_005956	*Bartonella henselae* str. Houston-1
NC_005955	*Bartonella quintana* str. Toulouse
NC_010161	*Bartonella tribocorum* CIP 105476
NC_007984	[*]*Baumannia cicadellinicola* str. Hc (*Homalodisca coagulata)*
NC_005363	[*]*Bdellovibrio bacteriovorus* HD100
NC_010581	[*]*Beijerinckia indica* subsp. *indica* ATCC 9039
NC_012669	[*]*Beutenbergia cavernae* DSM 12333
NC_008618	[*]*Bifidobacterium adolescentis* ATCC 15703
NC_011835	*Bifidobacterium animalis* subsp. *lactis* AD011
NC_012814	*Bifidobacterium animalis* subsp. *lactis* Bl-04
NC_012815	*Bifidobacterium animalis* subsp. *lactis* DSM 10140
NC_004307	*Bifidobacterium longum* NCC2705
NC_010816	*Bifidobacterium longum* DJO10A
NC_011593	*Bifidobacterium longum* subsp. *infantis* ATCC 15697
NC_013454	[*]*Blattabacterium* sp. (*Blattella germanica*) str. Bge
NC_013418	*Blattabacterium* sp. (*Periplaneta americana*) str. BPLAN
NC_010645	*Bordetella avium* 197N
NC_002927	*Bordetella bronchiseptica* RB50
NC_002928	*Bordetella parapertussis* 12822
NC_002929	[*]*Bordetella pertussis* Tohama I
NC_010170	*Bordetella petrii* DSM 12804
NC_008277	*Borrelia afzelii* PKo
NC_001318	[*]*Borrelia burgdorferi* B13
NC_011728	*Borrelia burgdorferi* ZS7
NC_011229	*Borrelia duttonii* Ly
NC_006156	*Borrelia garinii* PBi
NC_010673	*Borrelia hermsii* DAH
NC_011244	*Borrelia recurrentis* A1
NC_008710	*Borrelia turicatae* 91E135
NC_013172	[*]*Brachybacterium faecium* DSM 4810
NC_012225	[*]*Brachyspira hyodysenteriae* WA1
NC_009485	[*]*Bradyrhizobium* sp. BTAi1
NC_004463	*Bradyrhizobium japonicum* USDA 110
NC_009445	*Bradyrhizobium* sp. ORS278
NC_012491	[*]*Brevibacillus brevis* NBRC 100599
NC_006932 (chromosome 1); NC_006933 (chromosome 2)	*Brucella abortus* bv. 1 str. 9-941
NC_010742 (chromosome 1); NC_010740	[*]*Brucella abortus* S19

(chromosome 2)	
NC_010103 (chromosome 1); NC_010104 (chromosome 2)	*Brucella canis* ATCC 23365
NC_003317 (chromosome 1); NC_003318 (chromosome 2)	*Brucella melitensis* bv. 1 str. 16M
NC_012441 (chromosome 1); NC_012442 (chromosome 2)	*Brucella melitensis* ATCC 23457
NC_007618 (chromosome 1); NC_007624 (chromosome 2)	*Brucella melitensis* biovar Abortus 2308
NC_013119 (chromosome 1); NC_013118 (chromosome 2)	*Brucella microti* CCM 4915
NC_009505 (chromosome 1); NC_009504 (chromosome 2)	*Brucella ovis* ATCC 25840
NC_004310 (chromosome I); NC_004311 (chromosome 2)	*Brucella suis* 1330
NC_010169 (chromosome 1); NC_010167 (chromosome 2)	*Brucella suis* ATCC 23445
NC_002528	[*]*Buchnera aphidicola* str. APS (*Acyrthosiphon*)
NC_011833	*Buchnera aphidicola* str. 5A (*Acyrthosiphon pisum*)
NC_008513	*Buchnera aphidicola* str. Cc (*Cinara cedri*)
NC_004061	*Buchnera aphidicola* str. Sg (*Schizaphis graminum*)
NC_011834	*Buchnera aphidicola* str. Tuc7 (*Acyrthosiphon pisum*)
NC_004545	*Buchnera aphidicola* str. Bp (*Baizongia pistaciae*)
NC_007510 (chromosome 1); NC_007511 (chromosome 2)	*Burkholderia* sp. 383
NC_008390<br>NC_008391<br>NC_008392	*Burkholderia ambifaria* AMMD
NC_010551 (chromosome 1); NC_010552 (chromosome 2); NC_010557 (chromosome 3)	*Burkholderia ambifaria* MC40-6
NC_008060 (chromosome 1); NC_008061 (chromosome 2); NC_008062 (chromosome 3)	*Burkholderia cenocepacia* AU 1054
NC_008542 (chromosome 1); NC_008543	*Burkholderia cenocepacia* HI2424

(chromosome 2); NC_008544 (chromosome 3)	
NC_011000 (chromosome 1); NC_011001 (chromosome 2); NC_011002 (chromosome 3)	*Burkholderia cenocepacia* J2315
NC_010508 (chromosome 1); NC_010515 (chromosome 2); NC_010512 (chromosome 3)	*Burkholderia cenocepacia* MC0-3
NC_012724 (chromosome 1); NC_012721 (chromosome 2)	*Burkholderia glumae* BGR1
NC_006348 (chromosome 1); NC_006349 (chromosome 2)	*Burkholderia mallei* ATCC 23344
NC_008836 (chromosome 1); NC_008835 (chromosome 2)	*Burkholderia mallei* NCTC 10229
NC_009080 (chromosome 1); NC_009079 (chromosome 2)	*Burkholderia mallei* NCTC 10247
NC_008785 (chromosome 1); NC_008784 (chromosome 2)	*Burkholderia mallei* SAVP1
NC_010084 (chromosome 1); NC_010086 (chromosome 2); NC_010087 (chromosome 3)	*Burkholderia multivorans* ATCC 17616 JGI
NC_010804 (chromosome 1); NC_010805 (chromosome 2); NC_010801 (chromosome 3)	*Burkholderia multivorans* ATCC 17616 Tohoku
NC_010622 (chromosome 1); NC_010623 (chromosome 2)	*Burkholderia phymatum* STM815
NC_010681 (chromosome 1); NC_010676 (chromosome 2)	*Burkholderia phytofirmans* PsJN
NC_009076 (chromosome 1); NC_009078 (chromosome 2)	*Burkholderia pseudomallei* 1106a
NC_007434 (chromosome 1); NC_007435 (chromosome 2)	*Burkholderia pseudomallei* 1710b

NC_009074 (chromosome 1); NC_009075 (chromosome 2)	*Burkholderia pseudomallei* 668
NC_006350 (chromosome 1); NC_006351 (chromosome 2)	*Burkholderia pseudomallei* K96243
NC_012695	**Burkholderia pseudomallei* MSHR346
NC_007651 (chromosome 1); NC_007650 (chromosome 2)	*Burkholderia thailandensis* E264
NC_009256 (chromosome 1); NC_009255 (chromosome 2); NC_009254 (chromosome 3)	*Burkholderia vietnamiensis* G4
NC_007951 (chromosome 1); NC_007952 (chromosome 2); NC_007953 (chromosome 3)	*Burkholderia xenovorans* LB400
NC_009437	**Caldicellulosiruptor saccharolyticus* DSM 8903
NC_009954	**Caldivirga maquilingensis* IC-167
NC_009802	*Campylobacter concisus* 13826
NC_009715	*Campylobacter curvus* 525.92
NC_008599	*Campylobacter fetus* subsp. *fetus* 82-40
NC_009714	*Campylobacter hominis* ATCC BAA-381
NC_002163	**Campylobacter jejuni* NCTC 11168
NC_008787	*Campylobacter jejuni* subsp. *jejuni* 81-176
NC_009839	*Campylobacter jejuni* subsp. *jejuni* 81116
NC_009707	*Campylobacter jejuni* subsp. *doylei* 269.97
NC_003912	*Campylobacter jejuni* RM1221
NC_012039	*Campylobacter lari* RM2100
NC_013194	*Candidatus* Accumulibacter phosphatis clade IIA str, UW-1
NC_010830	*Candidatus* Amoebophilus asiaticus 5a2
NC_011565	*Candidatus* Azobacteroides pseudotrichonymphae genomovar. CFP2
NC_005061	*Candidatus* Blochmannia floridanus
NC_007292	*Candidatus* Blochmannia pennsylvanicus str.BPEN
NC_008512	*Candidatus* Carsonella ruddii PV
NC_009943	*Candidatus* Desulfococcus oleovorans Hxd3
NC_010424	*Candidatus* Desulforudis audaxviator MP104C
NC_012751	*Candidatus* Hamiltonella defensa 5AT (*Acyrthosiphon pisum*)
NC_012960	*Candidatus* Hodgkinia cicadicola Dsem
NC_010482	*Candidatus* Korarchaeum cryptofilum OPF8
NC_008009	*Candidatus* Koribacter versatilis Ellin345
NC_012985	*Candidatus* Liberibacter asiaticus str. psy62
NC_009712	*Candidatus* Methanoregula boonei 6A8
NC_011832	*Candidatus* Methanosphaerula palustris E1-9c
NC_007205	*Candidatus* Pelagibacter ubique HTCC1062

NC_010544	*Candidatus* Phytoplasma australiense
NC_011047	*Candidatus* Phytoplasma mali
NC_008610	*Candidatus* Ruthia magnifica str. Cm (*Calyptogena magnifica*)
NC_010118	*Candidatus* Sulcia muelleri GWSS
NC_013123	*Candidatus* Sulcia muelleri SMDSEM
NC_009465	*Candidatus* Vesicomyosocius okutanii HA
NC_013162	[*]*Capnocytophaga ochracea* DSM 7271
NC_007503	[*]*Carboxydothermus hydrogenoformans* Z-2901
NC_013131	[*]*Catenulispora acidiphila* DSM 44928
NC_002696	[*]*Caulobacter crescentus* CB15
NC_011916	*Caulobacter crescentus* NA1000
NC_010338	*Caulobacter* sp. K31
NC_010995	[*]*Cellvibrio japonicus* Ueda107
NC_013132	[*]*Chitinophaga pinensis* DSM 2588
NC_002620	*Chlamydia muridarum* NIGG
NC_010287	*Chlamydia trachomatis* 434/Bu
NC_007429	[*]*Chlamydia trachomatis* A/HAR-13
NC_012687	*Chlamydia trachomatis* B/TZ1A828/OT
NC_000117	*Chlamydia trachomatis* D/UW-3/CX
NC_012686	*Chlamydia trachomatis* B/Jali20/OT
NC_010280	*Chlamydia trachomatis* L2b/UCH-1/proctitis
NC_004552	*Chlamydophila abortus* S26/3
NC_003361	*Chlamydophila caviae* GPIC
NC_007899	*Chlamydophila felis* Fe/C-56
NC_002179	*Chlamydophila pneumoniae* AR39
NC_000922	*Chlamydophila pneumoniae* CWL029
NC_002491	*Chlamydophila pneumoniae* J138
NC_005043	*Chlamydophila pneumoniae* TW-183
NC_011027	[*]*Chlorobaculum parvum* NCIB 8327
NC_007514	[*]*Chlorobium chlorochromatii* CaD3
NC_010803	*Chlorobium limicola* DSM 245
NC_007512	*Chlorobium luteolum* DSM 273
NC_010831	*Chlorobium phaeobacteroides* BS1
NC_008639	*Chlorobium phaeobacteroides* DSM 266
NC_002932	*Chlorobium tepidum* TLS
NC_011831	[*]*Chloroflexus aggregans* DSM 9485
NC_010175	*Chloroflexus aurantiacus* J-10-fl
NC_012032	*Chloroflexus* sp. Y-400-fl
NC_011026	[*]*Chloroherpeton thalassium* ATCC 35110
NC_005085	[*]*Chromobacterium violaceum* ATCC 12472
NC_007963	[*]*Chromohalobacter salexigens* DSM 3043
NC_009792	[*]*Citrobacter koseri* ATCC BAA-895
NC_009480	[*]*Clavibacter michiganensis* subsp. *michiganensis* NCPPB 382
NC_010407	*Clavibacter michiganensis* subsp. *Sepedonicus*
NC_003030	*Clostridium acetobutylicum* ATCC 824
NC_009617	*Clostridium beijerinckii* NCIMB 8052
NC_009697	*Clostridium botulinum* A str. ATCC 19397
NC_012563	*Clostridium botulinum* A2 str. Kyoto

NC_010520	*Clostridium botulinum* A3 str. Loch Maree
NC_009697	*Clostridium botulinum* A str, ATCC 19397
NC_009698	*Clostridium botulinum* A str. Hall
NC_010516	*Clostridium botulinum* B1 str. Okra
NC_012658	*Clostridium botulinum* Ba4 str. 657
NC_010674	*Clostridium botulinum* B str. Eklund 17B
NC_010723	*Clostridium botulinum* E3 str. Alaska E43
NC_009699	*Clostridium botulinum* F str. Langeland
NC_011898	*Clostridium cellulolyticum* H10
NC_009089	*Clostridium difficile* 630
NC_013315	*Clostridium difficile* CD196
NC_013316	[*]*Clostridium difficile* R20291
NC_009706	*Clostridium kluyveri* DSM 555
NC_011837	*Clostridium kluyveri* NBRC 12016
NC_008593	*Clostridium novyi* NT
NC_003366	*Clostridium perfringens* str. 13
NC_008261	*Clostridium perfringens* ATCC 13124
NC_008262	*Clostridium perfringens* SM101
NC_010001	*Clostridium phytofermentans* ISDg
NC_004557	*Clostridium tetani* E88
NC_009012	*Clostridium thermocellum* ATCC 27405
NC_003910	[*]*Colwellia psychrerythraea* 34H
NC_013446	[*]*Comamonas testosteroni* CNB-2
NC_011295	[*]*Coprothermobacter proteolyticus* DSM 5265
NC_012590	*Corynebacterium aurimucosum* ATCC 700975
NC_002935	[*]*Corynebacterium diphtheriae* NCTC 13129
NC_004369	*Corynebacterium efficiens* YS-314
NC_006958	*Corynebacterium glutamicum* ATCC 13032 Bielefeld
NC_003450	*Corynebacterium glutamicum* ATCC 13032 Kitasato
NC_009342	*Corynebacterium glutamicum* R
NC_007164	*Corynebacterium jeikeium* K411
NC_012704	*Corynebacterium kroppenstedtii* DSM 44385
NC_010545	*Corynebacterium urealyticum* DSM 7109
NC_002971	[*]*Coxiella burnetii* RSA 493
NC_011527	*Coxiella burnetii* CbuG_Q212
NC_011528	*Coxiella burnetii* CbuK_Q154
NC_009727	*Coxiella burnetii* Dugway 5J108-111
NC_010117	*Coxiella burnetii* RSA 331
NC_013282	[*]*Cronobacter turicensis*
NC_013170	[*]*Cryptobacterium curtum* DSM 15641
NC_010528	[*]*Cupriavidus taiwanensis* str. LMG19424
NC_007775	[*]*Synechococcus* sp. JA-3-3Ab
NC_007776	*Synechococcus* sp. JA-2-3B'a(2-13)
NC_010546	[*]*Cyanothece* sp. ATCC 51142
NC_011729	*Cyanothece* sp. PCC 7424
NC_011884	*Cyanothece* sp. PCC 7425
NC_011726	*Cyanothece* sp. PCC 8801
NC_013161	*Cyanothece* sp. PCC 8802

NC_008255	**Cytophaga hutchinsonii* ATCC 33406
NC_007298	**Dechloromonas aromatica* RCB
NC_009455	**Dehalococcoides* sp. BAV1
NC_007356	*Dehalococcoides* sp. CBDB1
NC_002936	*Dehalococcoides ethenogenes* 195
NC_013552	*Dehalococcoides* sp. VS
NC_012526	*Deinococcus deserti* VCD115
NC_008025	*Deinococcus geothermalis* DSM 11300
NC_001263 (chromosome 1); NC_001264 (chromosome 2)	**Deinococcus radiodurans*
NC_010002	**Delftia acidovorans* SPH-1
NC_011768	**Desulfatibacillum alkenivorans* AK-01
NC_011830	*Desulfitobacterium hafniense* DCB-2
NC_007907	*Desulfitobacterium hafniense* Y51
NC_012108	*Desulfobacterium autotrophicum* HRM2
NC_013223	**Desulfohalobium retbaense* DSM 5692
NC_013173	**Desulfomicrobium baculatum* DSM 4028
NC_006138	**Desulfotalea psychrophila* LSv54
NC_013216	**Desulfotomaculum acetoxidans* DSM 771
NC_009253	*Desulfotomaculum reducens* MI-1
NC_011883	**Desulfovibrio desulfuricans* subsp. *desulfuricans* str. ATCC 27774
NC_007519	*Desulfovibrio desulfuricans* G20
NC_012796	*Desulfovibrio magneticus* RS-1
NC_012881	*Desulfovibrio salexigens* DSM 2638
NC_008751	*Desulfovibrio vulgaris* subsp. *vulgaris* DP4
NC_002937	*Desulfovibrio vulgaris* subsp. *vulgaris* str. Hildenborough
NC_011769	*Desulfovibrio vulgaris* str. ‘Miyazaki F’
NC_011766	**Desulfurococcus kamchatkensis* 1221n
NC_011992	*Acidovorax ebreus TPSY
NC_009446	**Dichelobacter nodosus* VCS1703A
NC_012880	**Dickeya dadantii* Ech703
NC_012912	*Dickeya zeae* Ech1591
NC_011297	**Dictyoglomus thermophilum* H-6-12
NC_011661	*Dictyoglomus turgidum* DSM 6724
NC_009952	**Dinoroseobacter shibae* DFL 12
NC_013037	**Dyadobacter fermentans* DSM 18053
NC_012779	*Edwardsiella ictaluri* 93-146
NC_013508	**Edwardsiella tarda* EIB202
NC_013204	**Eggerthella lenta* DSM 2243
NC_007354	**Ehrlichia canis* str. Jake
NC_007799	*Ehrlichia chaffeensis* str. Arkansas
NC_006831	*Ehrlichia ruminantium* str. Gardel
NC_006832	*Ehrlichia ruminantium* str. Welgevonden (CIRAD)
NC_005295	*Ehrlichia ruminantium* str. Welgevonden (UPSA)
NC_010644	**Elusimicrobium minutum* Pei191
NC_009436	*Enterobacter* sp. 638

NC_009778	*Enterobacter sakazakii* ATCC BAA-894
NC_004668	[*]*Enterococcus faecalis* V583
NC_004547	[*]*Erwinia carotovora* subsp. *atroseptica* SCRI1043
NC_012214	*Erwinia pyrifoliae* Ep1/96
NC_010694	*Erwinia tasmaniensis* Et1/99
NC_007722	[*]*Erythrobacter litoralis* HTCC2594
NC_011601	*Escherichia coli* O127:H6 str. E2348/69
NC_008253	*Escherichia coli* 536
NC_011748	*Escherichia coli* 55989
NC_008563	*Escherichia coli* APEC O1
NC_012947	*Escherichia coli* BL21(DE3)
NC_012759	*Escherichia coli* BW2952
NC_012967	*Escherichia coli* B str. REL606
NC_004431	*Escherichia coli* CFT073
NC_010468	*Escherichia coli* ATCC 8739
NC_009801	*Escherichia coli* E24377A
NC_011745	*Escherichia coli* ED1a
NC_009800	*Escherichia coli* HS
NC_011741	*Escherichia coli* IAI1
NC_011750	*Escherichia coli* IAI39
NC_010473	*Escherichia coli* str. K-12 substr. DH10B
NC_000913	*Escherichia coli* str. K-12 substr. MG1655 chromosome
AC_000091	*Escherichia coli* str. K-12 substr. W3110
NC_013353	*Escherichia coli* O103:H2 str. 12009
NC_013364	*Escherichia coli* O111:H- str. 11128
NC_002695	*Escherichia coli* O157:H7 str. Sakai
NC_002655	[*]*Escherichia coli* O157:H7 EDL933
NC_011353	*Escherichia coli* O157:H7 str. EC4115
NC_013008	*Escherichia coli* O157:H7 str. TW14359
NC_013361	*Escherichia coli* O26:H11 str. 11368
NC_011742	*Escherichia coli* S88
NC_011415	*Escherichia coli* SE11
NC_010498	*Escherichia coli* SMS-3-5
NC_011751	*Escherichia coli* UMN026
NC_007946	*Escherichia coli* UTI89
NC_011740	*Escherichia fergusonii* ATCC 35469
NC_012778	*Eubacterium eligens* ATCC 27750
NC_012781	[*]*Eubacterium rectale* ATCC 33656
NC_012673	[*]*Exiguobacterium* sp. AT1b
NC_010556	*Exiguobacterium sibiricum* 255-15
NC_009718	[*]*Fervidobacterium nodosum* Rt17-B1
NC_013410	[*]*Fibrobacter succinogenes* subsp. *succinogenes* S85
NC_010376	[*]*Finegoldia magna* ATCC 29328
NC_013062	*Flavobacteriaceae bacterium* 3519-10
NC_009441	[*]*Flavobacterium johnsoniae* UW101
NC_009613	*Flavobacterium psychrophilum* JIP02/86
NC_010336	*Francisella philomiragia* subsp. *philomiragia* ATCC 25017
NC_008245	[*]*Francisella tularensis* subsp. *tularensis* FSC 198

NC_007880	*Francisella tularensis* subsp. *holarctica*
NC_009749	*Francisella tularensis* subsp. *holarctica* FTNF002-00
NC_008369	*Francisella tularensis* subsp. *holarctica* OSU18
NC_010677	*Francisella tularensis* subsp. *mediasiatica* FSC147
NC_008601	*Francisella tularensis* subsp. *novicida* U112
NC_006570	*Francisella tularensis* subsp. *tularensis* SCHU S4
NC_009257	*Francisella tularensis* subsp. *tularensis* WY96-3418
NC_008278	*Frankia alni* ACN14a
NC_007777	*Frankia* sp. CcI3
NC_009921	**Frankia* sp. EAN1pec
NC_003454	**Fusobacterium nucleatum* subsp. *nucleatum* ATCC 25586
NC_012489	**Gemmatimonas aurantiaca* T-27
NC_006510	*Geobacillus kaustophilus* HTA426
NC_009328	**Geobacillus thermodenitrificans* NG80-2
NC_012793	*Geobacillus* sp. WCH70
NC_013406	*Geobacillus* sp. Y412MC10
NC_013411	*Geobacillus* sp. Y412MC61
NC_011146	*Geobacter bemidjiensis* Bem
NC_011979	*Geobacter* sp. FRC-32
NC_010814	*Geobacter lovleyi* SZ
NC_012918	*Geobacter* sp. M21
NC_007517	**Geobacter metallireducens* GS-15
NC_002939	*Geobacter sulfurreducens* PCA
NC_009483	*Geobacter uraniumreducens* Rf4
NC_005125	**Gloeobacter violaceus* PCC 7421
NC_010125	**Gluconacetobacter diazotrophicus* PAl 5 FAPERJ
NC_011365	*Gluconacetobacter diazotrophicus* PAl 5 JGI
NC_006677	**Gluconobacter oxydans* 621H
NC_013441	**Gordonia bronchialis* DSM 43247
NC_008571	**Gramella forsetii* KT0803
NC_008343	**Granulobacter bethesdensis* CGDNIH1
NC_002940	*Haemophilus ducreyi* 35000HP
NC_000907	**Haemophilus influenzae* Rd Kw20
NC_007146	*Haemophilus influenzae* 86-028NP
NC_009566	*Haemophilus influenzae* PittEE
NC_009567	*Haemophilus influenzae* PittGG
NC_011852	*Haemophilus parasuis* SH0165
NC_008309	*Haemophilus somnus* 129PT
NC_010519	*Haemophilus somnus* 2336
NC_007645	**Hahella chejuensis* KCTC 2396
NC_013440	**Haliangium ochraceum* DSM 14365
NC_006396 (chromosome 1); NC_006397 (chromosome 2)	**Haloarcula marismortui* ATCC 43049
NC_010364	**Halobacterium salinarum* R1
NC_002607	*Halobacterium* sp NRC-1
NC_013202	**Halomicrobium mukohataei* DSM 12286
NC_008212	**Haloquadratum walsbyi* DSM 16790

NC_013158	*Halorhabdus utahensis* DSM 12940
NC_008789	**Halorhodospira halophila* SL1
NC_012029 (chromosome 1); NC_012028 (chromosome 2)	**Halorubrum lacusprofundi* ATCC 49239
NC_011899	**Halothermothrix orenii* H 168
NC_013422	**Halothiobacillus neapolitanus* c2
NC_008229	*Helicobacter acinonychis* str. Sheeba
NC_004917	*Helicobacter hepaticus* ATCC 51449
NC_000915	**Helicobacter pylori* 26695
NC_012973	*Helicobacter pylori* B38
NC_011333	*Helicobacter pylori* G27
NC_008086	*Helicobacter pylori* HPAG1
NC_000921	*Helicobacter pylori* J99
NC_011498	*Helicobacter pylori* P12
NC_010698	*Helicobacter pylori* Shi470
NC_010337	**Heliobacterium modesticaldum* Ice1
NC_009138	**Herminiimonas arsenicoxydans*
NC_009972	**Herpetosiphon aurantiacus* ATCC 23779
NC_012982	**Hirschia baltica* ATCC 49814
NC_011126	**Hydrogenobaculum* sp. Y04AAS1
NC_008818	**Hyperthermus butylicus* DSM 5456
NC_008358	**Hyphomonas neptunium* ATCC 15444
NC_006512	**Idiomarina loihiensis* L2TR
NC_009776	**Ignicoccus hospitalis* KIN4/I
NC_007802	**Jannaschia* sp. CCS1
NC_009659	**Janthinobacterium* sp. *Marseille*
NC_013174	**Jonesia denitrificans* DSM 20603
NC_013166	**Kangiella koreensis* DSM 16069
NC_009664	**Kineococcus radiotolerans* SRS30216
NC_011283	**Klebsiella pneumoniae* 342
NC_009648	*Klebsiella pneumoniae* subsp. *pneumoniae* MGH 78578
NC_012731	*Klebsiella pneumoniae* NTUH K2044
NC_010617	**Kocuria rhizophila* DC2201
NC_012785	**Kosmotoga olearia* TBF 19.5.1
NC_013169	**Kytococcus sedentarius* DSM 20547
NC_006814	**Lactobacillus acidophilus* NCFM
NC_008497	*Lactobacillus brevis* ATCC 367
NC_010999	*Lactobacillus casei* BL23
NC_008526	*Lactobacillus casei* ATCC 334
NC_008054	*Lactobacillus delbrueckii* subsp. *bulgaricus* ATCC 11842
NC_008529	*Lactobacillus delbrueckii* subsp. *bulgaricus* ATCC BAA-365
NC_010610	*Lactobacillus fermentum* IFO 3956
NC_008530	*Lactobacillus gasseri* ATCC 33323
NC_010080	*Lactobacillus helveticus* DPC 4571
NC_013504	*Lactobacillus johnsonii* FI9785
NC_005362	*Lactobacillus johnsonii* NCC 533
NC_004567	*Lactobacillus plantarum* WCFS1

NC_012984	*Lactobacillus plantarum* JDM1
NC_009513	*Lactobacillus reuteri* DSM 20016
NC_010609	*Lactobacillus reuteri* JCM 1112
NC_013198	*Lactobacillus rhamnosus* GG
NC_013199	*Lactobacillus rhamnosus* Lc 705
NC_007576	*Lactobacillus sakei* subsp. *sakei* 23K
NC_007929	*Lactobacillus salivarius* UCC118
NC_002662	**Lactococcus lactis* subsp. *lactis* Il1403
NC_009004	*Lactococcus lactis* subsp. *cremoris* MG1363
NC_008527	*Lactococcus lactis* subsp. *cremoris* SK11
NC_012559	**Laribacter hongkongensis* HLHK9
NC_008011	**Lawsonia intracellularis* PHE/MN1-00
NC_009494	**Legionella pneumophila* str. Corby
NC_006369	*Legionella pneumophila* str. Lens
NC_006368	*Legionella pneumophila* str. Paris
NC_002942	*Legionella pneumophila* subsp. *pneumophila* str. Philadelphia 1
NC_006087	**Leifsonia xyli* subsp. *xyli* str. CTCB07
NC_010842 (chromosome 1); NC_010845 (chromosome 2)	*Leptospira biflexa* serovar Patoc strain 'Patoc 1 (Ames)'
NC_010602 (chromosome 1); NC_010843 (chromosome 2)	*Leptospira biflexa* serovar Patoc strain 'Patoc 1 (Paris)'
NC_008510 (chromosome 1); NC_008511 (chromosome 2)	*Leptospira borgpetersenii* serovar Hardjo-bovis JB197
NC_008508 (chromosome 1); NC_008509 (chromosome 2)	*Leptospira borgpetersenii* serovar Hardjo-bovis L550
NC_005823 (chromosome 1); NC_005824 (chromosome 2)	**Leptospira interrogans* serovar Copenhageni str. Fiocruz L1-130
NC_004342 (chromosome 1); NC_004343 (chromosome 2)	*Leptospira interrogans* serovar Lai str. 56601
NC_010524	**Leptothrix cholodnii* SP-6
NC_013192	**Leptotrichia buccalis* DSM 1135
NC_010471	**Leuconostoc citreum* KM20
NC_008531	*Leuconostoc mesenteroides* subsp. *mesenteroides* ATCC 8293
NC_003212	*Listeria innocua* Clip11262
NC_013768<br>NC_013766	**Listeria monocytogenes* 08-5923
NC_002973	*Listeria monocytogenes* 08-5578
NC_012488	*Listeria monocytogenes* str. 4b F2365
NC_011660	*Listeria monocytogenes* Clip81459
NC_008555	*Listeria monocytogenes* HCC23
NC_010382	**Listeria welshimeri* serovar 6b str. SLCC5334
NC_011999	**Lysinibacillus sphaericus* C3-41
NC_008576	**Macrococcus caseolyticus* JCSC5402

NC_007626	***Magnetococcus* sp. MC-1
NC_006300	***Magnetospirillum magneticum* AMB-1
NC_008347	***Mannheimia succiniciproducens* MBEL55E
NC_008740	***Maricaulis maris* MCS10
NC_009654	***Marinobacter aquaeolei* VT8
NC_008254	***Marinomonas* sp. MWYL1
NC_002678	***Mesorhizobium* sp. BNC1
NC_009440	*Mesorhizobium loti* MAFF303099
AE000666	*Metallosphaera sedula* DSM 5348
NC_009515	***Methanobacterium thermoautotrophicum* str. Delta H
NC_013156	***Methanobrevibacter smithii* ATCC 35061
NC_013407	***Methanocaldococcus fervens* AG86
NC_007955	*Methanocaldococcus vulcanius* M7
NC_009635	***Methanococcoides burtonii* DSM 6242
NC_000909	*Methanococcus aeolicus* Nankai-3
NC_009135	***Methanococcus jannaschii* DSM 2661
NC_009975	*Methanococcus maripaludis* C5
NC_009637	*Methanococcus maripaludis* C6
NC_005791	*Methanococcus maripaludis* C7
NC_009634	*Methanococcus maripaludis* S2
NC_008942	*Methanococcus vannielii* SB
NC_009051	***Methanocorpusculum labreanum* Z
AE009439	***Methanoculleus marisnigri* JR1
NC_008553	***Methanopyrus kandleri* AV19
NC_003552	***Methanosaeta thermophila* PT
NC_007355	***Methanosarcina acetivorans* C2A
NC_003901	*Methanosarcina barkeri* str. fusaro
NC_007681	***Methanosarcina mazei* Go1
NC_007796	***Methanosphaera stadtmanae* DSM 3091
NC_010794	***Methanospirillum hungatei* JF-1
NC_008825	***Methylacidiphilum infernorum* V4
NC_007947	***Methylibium petroleiphilum* PM1
NC_010511	***Methylobacillus flagellatus* KT
NC_011757	***Methylobacterium* sp. 4-46
NC_012808	*Methylobacterium chloromethanicum* CM4
NC_012988	*Methylobacterium extorquens* AM1
NC_010172	*Methylobacterium extorquens* DM4
NC_011894	*Methylobacterium extorquens* PA1
NC_010725	*Methylobacterium nodulans* ORS 2060
NC_010505	*Methylobacterium populi* BJ001
NC_011666	*Methylobacterium radiotolerans* JCM 2831
NC_002977	***Methylocella silvestris* BL2
NC_012968	***Methylococcus capsulatus* str. Bath
NC_012969	***Methylotenera mobilis* JLW8
NC_012803	***Methylovorus* sp. SIP3-4
NC_010296	***Micrococcus luteus* NCTC 2665
NC_007644	***Microcystis aeruginosa* NIES 843
NC_010397	***Moorella thermoacetica* ATCC 39073

NC_008595	*Mycobacterium abscessus* ATCC 19977T
NC_002944	*Mycobacterium avium* 104
NC_002945	*Mycobacterium avium paratuberculosis* K-10
NC_008769	*Mycobacterium bovis* AF2122/97
NC_012207	*Mycobacterium bovis* BCG str. Pasteur 1173P2
NC_009338	*Mycobacterium bovis* BCG str. Tokyo 172
NC_009077	*Mycobacterium gilvum* PYR-GCK
NC_008705	*Mycobacterium* sp. JLS
NC_002677	*Mycobacterium* sp. KMS
NC_011896	*Mycobacterium leprae* TN
NC_010612	*Mycobacterium leprae* Br4923
NC_008146	*Mycobacterium marinum* M
NC_008596	*Mycobacterium* sp. MCS
NC_002755	*Mycobacterium smegmatis* str. MC2 155
NC_009565	**Mycobacterium tuberculosis* CDC1551
NC_009525	*Mycobacterium tuberculosis* F11
NC_000962	*Mycobacterium tuberculosis* H37Ra
NC_012943	*Mycobacterium tuberculosis* H37Rv
NC_008611	*Mycobacterium tuberculosis* KZN 1435
NC_008726	*Mycobacterium ulcerans* Agy99
NC_008095	*Mycobacterium vanbaalenii* PYR-1
NC_013235	**Myxococcus xanthus* DK 1622
AE017199	**Nakamurella multipartita* DSM 44233
NC_010718	**Nanoarchaeum equitans* Kin4-M
NC_007426	**Natranaerobius thermophilus* JW/NM-WN-LF
NC_012115	**Natronomonas pharaonis* DSM 2160
NC_002946	**Nautilia profundicola* AmH
NC_011035	**Neisseria gonorrhoeae* FA 1090
NC_010120	*Neisseria gonorrhoeae* NCCP11945
NC_013016	*Neisseria meningitidis* 053442
NC_008767	*Neisseria meningitidis* alpha14
NC_003112	*Neisseria meningitidis* FAM18
NC_003116	*Neisseria meningitidis* MC58
NC_013009	*Neisseria meningitidis* Z2491
NC_007798	**Neorickettsia risticii* str. Illinois
NC_009662	*Neorickettsia sennetsu* str. Miyayama
NC_007964	**Nitratiruptor* sp. SB155-2
NC_007406	**Nitrobacter hamburgensis* X14
NC_007484	*Nitrobacter winogradsky*i Nb-255
NC_004757	**Nitrosococcus oceani* ATCC 19707
NC_008344	**Nitrosomonas europaea* ATCC 19718
NC_010085	*Nitrosomonas eutropha* C91
NC_007614	**Nitrosopumilus maritimus* SCM1
NC_006361	**Nitrosospira multiformis* ATCC 25196
NC_008699	**Nocardia farcinica* IFM10152
NC_010628	*Nocardioides* sp. JS614
NC_003272	**Nostoc punctiforme* PCC 73102
NC_007794	*Nostoc* sp PCC 7120

NC_004193	*Novosphingobium aromaticivorans* DSM 12444
NC_009667 (chromosome 1); NC_009668 (chromosome 2)	**Oceanobacillus iheyensis* HTE831
NC_008528	**Ochrobactrum anthropi* ATCC 49188
NC_011386	**Oenococcus oeni* PSU-1
NC_005303	**Oligotropha carboxidovorans* OM5
NC_010571	*Onion yellows phytoplasma* OY-M
NC_009488	**Opitutus terrae* PB90-1
NC_010793	**Orientia tsutsugamushi* Boryong
NC_012914	*Orientia tsutsugamushi* str. Ikeda
NC_009615	**Paenibacillus* sp. JDR 2
NC_005861	**Parabacteroides distasonis* ATCC 8503
NC_008686 (chromosome 1); NC_008686 (chromosome 2)	**Candidatus* Protochlamydia amoebophila UWE25
NC_009719	**Paracoccus denitrificans* PD1222
NC_002663	**Parvibaculum lavamentivorans* DS-1
NC_012917	**Pasteurella multocida* subsp. *multocida* str. Pm70
NC_013421	**Pectobacterium carotovorum* subsp. *carotovorum* PC1
NC_008525	*Pectobacterium wasabiae* WPP163
NC_013061	**Pediococcus pentosaceus* ATCC 25745
NC_007498	**Pedobacter heparinus* DSM 2366
NC_008609	*Pelobacter carbinolicus* DSM 2380
NC_011060	*Pelobacter propionicus* DSM 2379
NC_009454	**Pelodictyon phaeoclathratiforme* BU-1
NC_012440	**Pelotomaculum thermopropionicum* SI
NC_010003	**Persephonella marina* EX-H1
NC_011144	**Petrotoga mobilis* SJ95
NC_006370 (chromosome 1); NC_006371 (chromosome 2)	**Phenylobacterium zucineum* HLK1
NC_012962	**Photobacterium profundum* SS9
NC_005126	**Photorhabdus asymbiotica*
NC_005877	*Photorhabdus luminescens* subsp. *laumondii* TTO1
NC_013720	**Picrophilus torridus* DSM 9790
NC_007948	**Pirellula staleyi* DSM 6068
NC_008781	**Polaromonas* sp. JS666
NC_009379	*Polaromonas naphthalenivorans* CJ2
NC_010531	**Polynucleobacter necessarius* subsp. *asymbioticus* QLW-P1DMWA-1
NC_010729	*Polynucleobacter necessarius* subsp. *necessarius* STIR1
NC_002950	**Porphyromonas gingivalis* ATCC 33277
NC_008816	*Porphyromonas gingivalis* W83
NC_005042	**Prochlorococcus marinus* str. AS9601
NC_005072	*Prochlorococcus marinus* subsp. *marinus* CCMP1375
NC_005071	*Prochlorococcus marinus* subsp. *pastoris* str, CCMP1986
NC_009976	*Prochlorococcus marinus* str. MIT9313
NC_009840	*Prochlorococcus marinus* str. MIT 9211

NC_009091	*Prochlorococcus marinus* str. MIT 9215
NC_008820	*Prochlorococcus marinus* str. MIT 9301
NC_007577	*Prochlorococcus marinus* str. MIT 9303
NC_008817	*Prochlorococcus marinus* str. MIT 9312
NC_008819	*Prochlorococcus marinus* str. MIT 9515
NC_007335	*Prochlorococcus marinus* str. NATL1A
NC_006085	*Prochlorococcus marinus* str. NATL2A
NC_011059	[*]*Propionibacterium acnes* KPA171202
NC_009337	[*]*Prosthecochloris aestuarii* DSM 271
NC_010554	*Prosthecochloris vibrioformis* DSM 265
NC_008228	[*]*Proteus mirabilis* HI4320
NC_007481 (chromosome 1); NC_007482 (chromosome 2)	[*]*Pseudoalteromonas atlantica* T6c
NC_002516	*Pseudoalteromonas haloplanktis* TAC125
NC_011770	[*]*Pseudomonas aeruginosa* PAO1
NC_009656	*Pseudomonas aeruginosa* LESB58
NC_008463	*Pseudomonas aeruginosa* PA7
NC_008027	*Pseudomonas aeruginosa* UCBPP-PA14
NC_004129	*Pseudomonas entomophila* L48
NC_007492	*Pseudomonas fluorescens* Pf-5
NC_012660	*Pseudomonas fluorescens* Pf0-1
NC_009439	*Pseudomonas fluorescens* SBW25
NC_009512	*Pseudomonas mendocina* ymp
NC_010322	*Pseudomonas putida* F1
NC_002947	*Pseudomonas putida* GB-1
NC_010501	*Pseudomonas putida* KT2440
NC_009434	*Pseudomonas putida* W619
NC_005773	*Pseudomonas stutzeri* A1501
NC_007005	*Pseudomonas syringae* pv. *phaseolicola* 1448A
NC_004578	*Pseudomonas syringae* pv *syringae* B728a
NC_007204	*Pseudomonas syringae* pv. *tomato* str. DC3000
NC_007969	[*]*Psychrobacter arcticum* 273-4
NC_009524	*Psychrobacter cryohalolentis* K5
NC_008709	*Psychrobacter* sp. PRwf-1
NC_003364	*Psychromonas ingrahamii* 37
NC_009376	[*]*Pyrobaculum aerophilum* str. IM2
NC_009073	*Pyrobaculum arsenaticum* DSM 13514
NC_008701	*Pyrobaculum calidifontis* JCM 11548
NC_000868	*Pyrobaculum islandicum* DSM 4184
NC_003413	*Pyrococcus abyssi* GE5
NC_000961	[*]*Pyrococcus furiosus* DSM 3638
NC_008313 (chromosome 1); NC_008314 (chromosome 2)	*Pyrococcus horikoshii* OT3
NC_007347 (chromosome 1); NC_007348 (chromosome 2)	*Ralstonia eutropha* H16

NC_007973 (chromosome 1); NC_007974 (chromosome 2)	*Ralstonia eutropha* JMP134
NC_012856 (chromosome 1); NC_012857 (chromosome 2)	**Ralstonia metallidurans* CH34
NC_010682 (chromosome 1); NC_010678 (chromosome 2)	*Ralstonia pickettii* 12D
NC_003295	*Ralstonia pickettii* 12J
NC_010168	*Ralstonia solanacearum* GMI1000
NC_007761	**Renibacterium salmoninarum* ATCC 33209
NC_010994	**Rhizobium etli* CFN 42
NC_008380	*Rhizobium etli* CIAT 652
NC_012850	*Rhizobium leguminosarum* bv. *viciae* 3841
NC_011369	*Rhizobium leguminosarum* bv. *trifolii* WSM1325
NC_012587	*Rhizobium leguminosarum* bv. *trifolii* WSM2304
NC_007493 (chromosome 1); NC_007494 (chromosome 2)	*Rhizobium* sp. NGR234
NC_009428	*Rhodobacter sphaeroides* 2.4.1
NC_009049 (chromosome 1); NC_009050 (chromosome 2)	**Rhodobacter sphaeroides* ATCC 17025
NC_011963 (chromosome 1); NC_011958 (chromosome 2)	*Rhodobacter sphaeroides* ATCC 17029
NC_012490	*Rhodobacter sphaeroides* KD131
NC_008268	*Rhodococcus erythropolis* PR4
NC_012522	**Rhodococcus jostii* RHA1
NC_007908	*Rhodococcus opacus* B4
NC_008435	**Rhodoferax ferrireducens* T118
NC_007925	**Rhodopseudomonas palustris* BisA53
NC_007958	*Rhodopseudomonas palustris* BisB18
NC_005296	*Rhodopseudomonas palustris* BisB5
NC_007778	*Rhodopseudomonas palustris* CGA009
NC_011004	*Rhodopseudomonas palustris* HaA2
NC_011420	*Rhodopseudomonas palustris* TIE-1
NC_007643	**Rhodospirillum centenum* SW
NC_013501	*Rhodospirillum rubrum* ATCC 11170
NC_012633	**Rhodothermus marinus* DSM 4252
NC_009881	*Rickettsia africae* ESF-5
NC_009883	*Rickettsia akari* str. Hartford
NC_007940	*Rickettsia bellii* OSU 85-389
NC_009879	*Rickettsia bellii* RML369-C
NC_003103	*Rickettsia canadensis* str. McKiel
NC_007109	*Rickettsia conorii* str. Malish 7
NC_009900	*Rickettsia felis* URRWXCal2
NC_012730	*Rickettsia massiliae* MTU5

NC_000963	*Rickettsia peacockii* str. Rustic
NC_010263	**Rickettsia prowazekii* str. Madrid E
NC_009882	*Rickettsia rickettsii* str. Iowa
NC_006142	*Rickettsia rickettsii* str. ‘Sheila Smith’
NC_013222	*Rickettsia typhi* str. wilmington
NC_009767	**Robiginitalea biformata* HTCC2501
NC_009523	**Roseiflexus castenholzii* DSM 13941
NC_008209	*Roseiflexus* sp. RS-1
NC_008148	**Roseobacter denitrificans* OCh 114
NC_003911	**Rubrobacter xylanophilus* DSM 9941
NC_013159	**Ruegeria pomeroyi* DSS-3
NC_007912	**Saccharomonospora viridis* DSM 43017
NC_009142	**Saccharophagus degradans* 2-40
NC_007677	**Saccharopolyspora erythraea* NRRL 2338
NC_009953	**Salinibacter ruber* DSM 13855
NC_009380	**Salinispora arenicola* CNS-205
NC_010067	*Salinispora tropica* CNB-440
NC_006905 (SC-B67)	*Salmonella enterica* subsp. *arizonae* serovar 62:z4, z23:--
NC_006511	**Salmonella enterica* subsp. *Choleraesuis*
NC_011149	*Salmonella enterica* subsp. *enterica* serovar *Paratypi A* str. ATCC 9150
NC_011205	*Salmonella enterica* subsp. *enterica* serovar Agona str. SL483
NC_011294	*Salmonella enterica* subsp. *enterica* serovar Dublin str. CT 02021853
NC_011274	*Salmonella enterica* subsp. *enterica* serovar Enteritidis str. P125109
NC_011083	*Salmonella enterica* subsp. *enterica* serovar Gallinarum str. 287/91
NC_011080	*Salmonella enterica* subsp. *enterica* serovar Heidelberg str. SL476
NC_011147	*Salmonella enterica* subsp. *enterica* serovar Newport str. SL254
NC_010102	*Salmonella enterica* subsp. *enterica* serovar Paratyphi A str. AKU 12601
NC_012125	*Salmonella enterica* subsp. *enterica* serovar Paratyphi B str. SPB7
NC_011094	*Salmonella enterica* subsp. *enterica* serovar Paratyphi C strain RKS4594
NC_004631	*Salmonella enterica* subsp. *enterica* serovar Schwarzengrund str. CVM19633
NC_003198	*Salmonella enterica* subsp. *enterica* serovar Typhi str. Ty2
NC_003197	*Salmonella enterica* subsp. *enterica* serovar Typhi str CT18
NC_013517	*Salmonella enterica* subsp. *enterica* serovar *typhimurium* str. LT2
NC_009832	**Sebaldella termitidis* ATCC 33386
NC_008700	**Serratia proteamaculans* 568
NC_008577	*Shewanella amazonensis* SB2B
NC_009052	*Shewanella* sp. ANA-3
NC_009665	*Shewanella baltica* OS155
NC_009997	*Shewanella baltica* OS185

NC_011663	*Shewanella baltica* OS195
NC_007954	*Shewanella baltica* OS223
NC_008345	[*]*Shewanella denitrificans* OS217
NC_010334	*Shewanella frigidimarina* NCIMB 400
NC_009092	*Shewanella halifaxensis* HAW-EB4
NC_008321	*Shewanella loihica* PV-4
NC_008322	*Shewanella* MR-4
NC_004347	*Shewanella* MR-7
NC_009901	*Shewanella oneidensis* MR-1
NC_011566	*Shewanella pealeana* ATCC 700345
NC_009438	*Shewanella piezotolerans* WP3
NC_009831	*Shewanella putrefaciens* CN-32
NC_008750	*Shewanella sediminis* HAW-EB3
NC_010506	*Shewanella* sp. W3-18-1
NC_010658	*Shewanella woodyi* ATCC 51908
NC_007613	[*]*Shigella boydii* CDC 3083-94
NC_007606	*Shigella boydii* Sb227
NC_004337 (str. 301)	*Shigella dysenteriae* Sd197
NC_004741	*Shigella flexneri* 2a
NC_008258	*Shigella flexneri* 2a str. 2457T
NC_007384	*Shigella flexneri* 5 str. 8401
CP000377	*Shigella sonnei* Ss046
NC_009636	[*]*Silicibacter* TM1040
NC_003047	[*]*Sinorhizobium medicae* WSM419
NC_013165	*Sinorhizobium meliloti* 1021
NC_007712	[*]*Slackia heliotrinireducens* DSM 20476
NC_008536	[*]*Sodalis glossinidius* str. '*morsitans'*
NC_010162	[*]*Solibacter usitatus* Ellin6076
NC_013523(chromosome I); NC_013524 (chromosome II)	[*]*Sorangium cellulosum* 'So ce 56'
NC_009511	[*]*Sphaerobacter thermophilus* DSM 20745
NC_008048	[*]*Sphingomonas wittichii* RW1
NC_002952	[*]*Sphingopyxis alaskensis* RB2256
NC_002953	[*]*Staphylococcus aureus* subsp. *aureus* MRSA252
NC_002951	*Staphylococcus aureus* subsp. *aureus* MSSA476
NC_013450	*Staphylococcus aureus* subsp. *aureus* COL
NC_009632	*Staphylococcus aureus* ED98
NC_009487	*Staphylococcus aureus* subsp. *aureus* JH1
NC_009782	*Staphylococcus aureus* subsp. *aureus* JH9
NC_002758	*Staphylococcus aureus* subsp. *aureus* Mu3
NC_003923	*Staphylococcus aureus* subsp. *aureus* Mu50
NC_002745	*Staphylococcus aureus* subsp. *aureus* MW2
NC_007795	*Staphylococcus aureus* subsp. *aureus* N315
NC_009641	*Staphylococcus aureus* subsp. *aureus* NCTC 8325
NC_007622	*Staphylococcus aureus* subsp. *aureus* str. Newman
NC_007793	*Staphylococcus aureus* RF122
NC_010079	*Staphylococcus aureus* subsp. *aureus* USA300_FPR3757

NC_012121	*Staphylococcus aureus* subsp. *aureus* USA300_TCH1516
NC_004461	*Staphylococcus carnosus* subsp. *carnosus* TM300
NC_002976	*Staphylococcus epidermidis* ATCC 12228
NC_007168	*Staphylococcus epidermidis* RP62A
NC_007350	*Staphylococcus haemolyticus* JCSC1435
NC_009033	*Staphylococcus saprophyticus* subsp. *Saprophyticus* ATCC 15305
NC_010943	*Staphylothermus marinus* F1
NC_011071	**Stenotrophomonas maltophilia* K279a
NC_013515	*Stenotrophomonas maltophilia* R551-3
NC_004116	**Streptobacillus moniliformis* DSM 12112
NC_007432	*Streptococcus agalactiae* 2603V/R
NC_004368	*Streptococcus agalactiae* A909
NC_012891	*Streptococcus agalactiae* NEM316
NC_012471	*Streptococcus dysgalactiae* subsp. *equisimilis* GGS_124
NC_012470	*Streptococcus equi* subsp. *equi* 4047
NC_011134	*Streptococcus equi* subsp. *zooepidemicus*
NC_009785	*Streptococcus equi* subsp. *zooepidemicus* MGCS10565
NC_004350	*Streptococcus gordonii* str. Challis substr. CH1
NC_012468	*Streptococcus mutans* UA159
NC_011900	*Streptococcus pneumoniae* 70585
NC_010582	*Streptococcus pneumoniae* ATCC 700669
NC_008533	*Streptococcus pneumoniae* CGSP14
NC_011072	*Streptococcus pneumoniae* D39
NC_010380	*Streptococcus pneumoniae* G54
NC_012466	*Streptococcus pneumoniae* Hungary19A-6
NC_012467	*Streptococcus pneumoniae* JJA
NC_003098	*Streptococcus pneumoniae* P1031
NC_012469	*Streptococcus pneumoniae* R6
NC_003028	*Streptococcus pneumoniae* Taiwan19F-14
NC_002737	**Streptococcus pneumoniae* TIGR4
NC_009332	*Streptococcus pyogenes* M1 GAS
NC_008022	*Streptococcus pyogenes* str. Manfredo
NC_006086	*Streptococcus pyogenes* MGAS10270
NC_008024	*Streptococcus pyogenes* MGAS10394
NC_008023	*Streptococcus pyogenes* MGAS10750
NC_004070	*Streptococcus pyogenes* MGAS2096
NC_007297	*Streptococcus pyogenes* MGAS315
NC_007296	*Streptococcus pyogenes* MGAS5005
NC_003485	*Streptococcus pyogenes* MGAS6180
NC_008021	*Streptococcus pyogenes* MGAS8232
NC_011375	*Streptococcus pyogenes* MGAS9429
NC_004606	*Streptococcus pyogenes* NZ131
NC_009009	*Streptococcus pyogenes* SSI-1
NC_009442	*Streptococcus sanguinis* SK36
NC_009443	*Streptococcus suis* 05ZYH33
NC_012926	*Streptococcus suis* 98HAH33
NC_012925	*Streptococcus suis* BM407

NC_012924	*Streptococcus suis* P1/7
NC_006449	*Streptococcus suis* SC84
NC_008532	*Streptococcus thermophilus* CNRZ1066
NC_006448	*Streptococcus thermophilus* LMD-9
NC_012004	*Streptococcus thermophilus* LMG 18311
NC_003155	*Streptococcus uberis* 0140J
NC_003888	*Streptomyces avermitilis* MA-4680
NC_010572	**Streptomyces coelicolor* A3(2)
NC_007181	*Streptomyces griseus* subsp. *griseus* NBRC 13350
NC_012589	*Sulfolobus acidocaldarius* DSM 639
NC_012588	*Sulfolobus islandicus* L.S.2.15
NC_012632	*Sulfolobus islandicus* M.14.25
NC_012726	*Sulfolobus islandicus* M.16.27
NC_012622	*Sulfolobus islandicus* M.16.4
NC_012623	*Sulfolobus islandicus* Y.G.57.14
NC_002754	*Sulfolobus islandicus* Y.N.15.51
NC_003106	*Sulfolobus solfataricus* P2
NC_012438	**Sulfolobus tokodaii* str. 7
NC_010730	**Sulfurihydrogenibium azorense* Az-Fu1
NC_013512	*Sulfurihydrogenibium* sp. YO3AOP1
NC_009663	**Sulfurospirillum deleyianum* DSM 6946
NC_006177	**Sulfurovum* sp. NBC37-1
NC_008319	**Symbiobacterium thermophilum* IAM14863
NC_007516	**Synechococcus* sp. CC9311
NC_007513	*Synechococcus* sp. CC9605
NC_006576	*Synechococcus* sp. CC9902
NC_007604	*Synechococcus elongatus* PCC 6301
NC_010475	*Synechococcus elongatus* PCC 7942
NC_009482	*Synechococcus* sp. PCC 7002
	Synechococcus sp. RCC307
NC_009481	*Synechococcus* sp. WH8102
NC_000911	*Synechococcus* sp. WH 7803
NC_008554	*Synechocystis* sp. PCC6803
NC_008346	**Syntrophobacter fumaroxidans* MPOB
NC_007759	**Syntrophomonas wolfei* subsp. *wolfei* str. Goettingen
NC_012997	**Syntrophus aciditrophicus* SB
NC_011662	**Teredinibacter turnerae* T7901
NC_013522	**Thauera* sp. MZ1T
NC_010321	**Thermanaerovibrio acidaminovorans* DSM 6589
NC_003869	**Thermoanaerobacter pseudethanolicus* ATCC 33223
NC_010320	*Thermoanaerobacter tengcongensis* MB4
NC_013525 (Chromosome I); NC_013526 (Chromosome II)	*Thermoanaerobacter* sp. X514
NC_007333	**Thermobaculum terrenum* ATCC BAA-798
NC_012804	**Thermobifida fusca* YX
NC_006624	**Thermococcus gammatolerans* EJ3

NC_011529	*Thermococcus kodakaraensis* KOD1
NC_012883	*Thermococcus onnurineus* NA1
NC_011296	*Thermococcus sibiricus* MM 739
NC_008698	**Thermodesulfovibrio yellowstonii* DSM 11347
NC_011959	**Thermofilum pendens* Hrk 5
NC_013510	**Thermomicrobium roseum* DSM 5159
NC_002578	**Thermomonospora curvata* DSM 43183
NC_002689	**Thermoplasma acidophilum* DSM 1728
NC_010525	*Thermoplasma volcanium* GSS1
NC_011653	**Thermoproteus neutrophilus* V24Sta
NC_009616	**Thermosipho africanus* TCF52B
NC_004113	*Thermosipho melanesiensis* BI429
NC_009828	**Thermosynechococcus elongates* BP-1
NC_000853	*Thermotoga lettingae* TMO
NC_011978	**Thermotoga maritime* MSB8
NC_009486	*Thermotoga neapolitana* DSM 4359
NC_010483	*Thermotoga petrophila* RKU-1
NC_005835	*Thermotoga* sp. RQ2
NC_006461	**Thermus thermophilus* HB27
NC_011901	*Thermus thermophilus* HB8
NC_007404	**Thioalkalivibrio* sp. HL-EbGR7
NC_007520	**Thiobacillus denitrificans* ATCC 25259
NC_007404	**Thiomicrospira crunogena* XCL-2
NC_012691	*Thiomicrospira denitrificans* ATCC 25259
NC_002967	**Tolumonas auensis* DSM 9187
NC_000919 (Nichols)	*Treponema denticola* ATCC 35405
NC_010741	**Treponema pallidum* subsp. *pallidum* str. Nichols
NC_008312	*Treponema pallidum* subsp. *pallidum* SS14
NC_004551	**Trichodesmium erythraeum* IMS101
NC_004572	**Tropheryma whipplei* TW08/27
NC_009464	*Tropheryma whipplei* str. Twist
NS_000191	*uncultured methanogenic archaeon RC-I
NC_012791 (Chromosome I) ; NC_012792 (Chromosome II)	*uncultured Termite group 1 bacterium phylotype Rs D17
NC_013520	**Variovorax paradoxus* S110
NC_008786	**Veillonella parvula* DSM 2008
NC_002505 (chromosome 1); NC_002506 (chromosome II)	**Verminephrobacter eiseniae* EF01-2
NC_012578 (chromosome I)	**Vibrio cholerae* O1 biovar Eltor str. N16961
NC_012668 (chromosome I); NC_012667 (Chromosome II)	*Vibrio cholerae* M66-2
NC_009456 (Chromosome I); NC_009457	*Vibrio cholerae* MJ-1236

(Chromosome II)	
NC_013456 (Chromosome I); NC_013457 (Chromosome II)	*Vibrio cholerae* O395
NC_006840 (chromosome I); NC_006841	*Vibrio* sp. Ex25
NC_011184 (chromosome I); NC_011186 (chromosome II)	*Vibrio fischeri* ES114
NC_009783 (chromosome I); NC_009784 (chromosome II)	*Vibrio fischeri* MJ11
NC_004603 (chromosome 1); NC_004605 (chromosome II)	*Vibrio harveyi* ATCC BAA-1116
NC_011753 (chromosome 1); NC_011744 (chromosome II)	*Vibrio parahaemolyticus* RIMD 2210633
NC_004459 (chromosome I); NC_004460 (chromosome II)	*Vibrio splendidus* LGP32
NC_005139 (chromosome I); NC_005140 (chromosome II)	*Vibrio vulnificus* CMCP6
NC_004344	*Vibrio vulnificus* YJ016
NC_006833	[*]*Wigglesworthia glossinidia* endosymbiont of *Glossina brevipalpis*
NC_010981	[*]*Wolbachia* endosymbiont of *Brugia malayi* TRS
NT_037436	*Wolbachia* endosymbiont of *Culex quinquefasciatus* Pel
NC_012416	*Wolbachia* endosymbiont of *Drosophila melanogaster*
NC_005090	*Wolbachia* sp. wRi
NC_009720	*Wolinella succinogenes* DSM 1740
NC_007086	*Xanthobacter autotrophicus* Py2
NC_003902	*Xanthomonas campestris* pv. *campestris* str. 8004
NC_010688	*Xanthomonas campestris* pv. *campestris* str.ATCC 33913
NC_007508	*Xanthomonas campestris* pv. *campestris* str. B100
NC_003919	*Xanthomonas campestris* pv. *vesicatoria* str. 85-10
NC_006834	Xanthomonas axonopodis pv. *citri* str. 306
NC_007705	*Xanthomonas oryzae* pv. *oryzae* KACC10331
NC_010717	*Xanthomonas oryzae* pv. *oryzae* MAFF 311018
NC_013530	[*]*Xanthomonas oryzae* pv. *oryzae* PXO99A
NC_002488	[*]*Xylanimonas cellulosilytica* DSM 15894
NC_010513	[*]*Xylella fastidiosa* 9a5c
NC_010577	*Xylella fastidiosa* M12
NC_004556	*Xylella fastidiosa* M23
NC_008800	*Xylella fastidiosa* Temecula1
NC_010159	[*]*Yersinia enterocolitica* subsp. *enterocolitica* 8081
NC_008150	*Yersinia pestis* Angola

NC_005810	*Yersinia pestis* Antiqua
NC_003143	*Yersinia pestis* biovar Microtus str. 91001
NC_004088	*Yersinia pestis* CO92
NC_008149	*Yersinia pestis* KIM 10
NC_009381	*Yersinia pestis* Nepal516
NC_006155	*Yersinia pestis* Pestoides F
NC_009708	*Yersinia pseudotuberculosis* IP32953
NC_010634	*Yersinia pseudotuberculosis* IP 31758
NC_010465	*Yersinia pseudotuberculosis* PB1/+
NC_013355	*Yersinia pseudotuberculosis* YPIII
NC_006526	[*]*Zymomonas mobilis* subsp. *mobilis* NCIMB 11163

[*] indicates that the genome was selected for Markov modeling of OSC frequencies.

LEGENDS TO SUPPLEMENTARY FIGURES

Supplementary figure S1. Scree plot of the principal component analysis (PCA) on the relative codon frequencies of OSCs in both alternate reading frames, showing that the first principal axis explained the majority of variance in the data.

Supplementary figure S2. Biplot of the PCA results, showing that TAG usage bias in frame +3 (TAG2) is not significantly associated with the first 2 principal component axes. OSC usage bias in the +2 frame appears to be more significantly correlated with the first principal component axis as compared to the OSC usage bias in the +3 frame.

Supplementary figure S3. Diagram depicting the relationships between the various Markov models used in the present analysis. Directly comparable models are linked with lines.

Suppl. figure **S1**

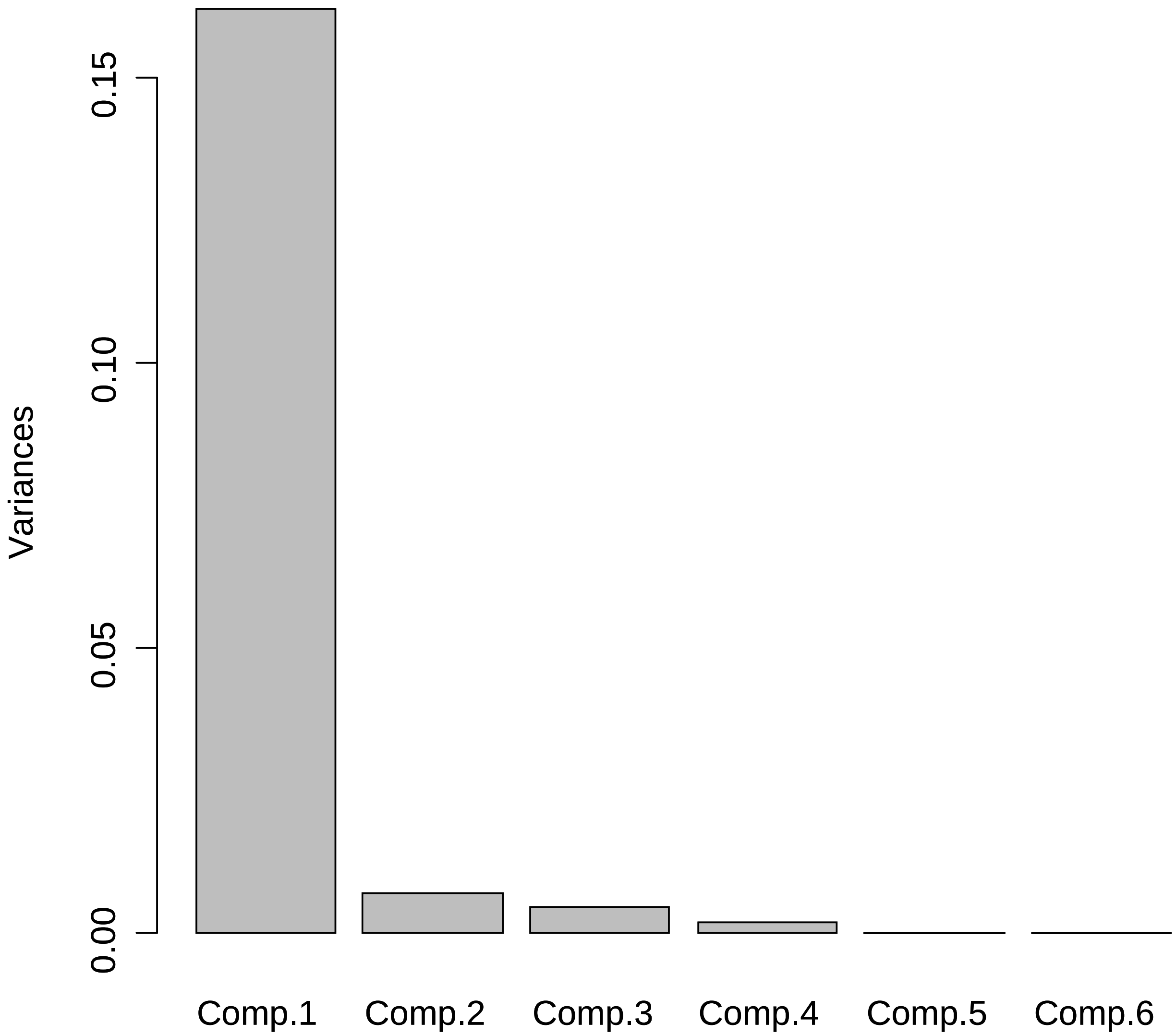

Suppl. figure **S2**

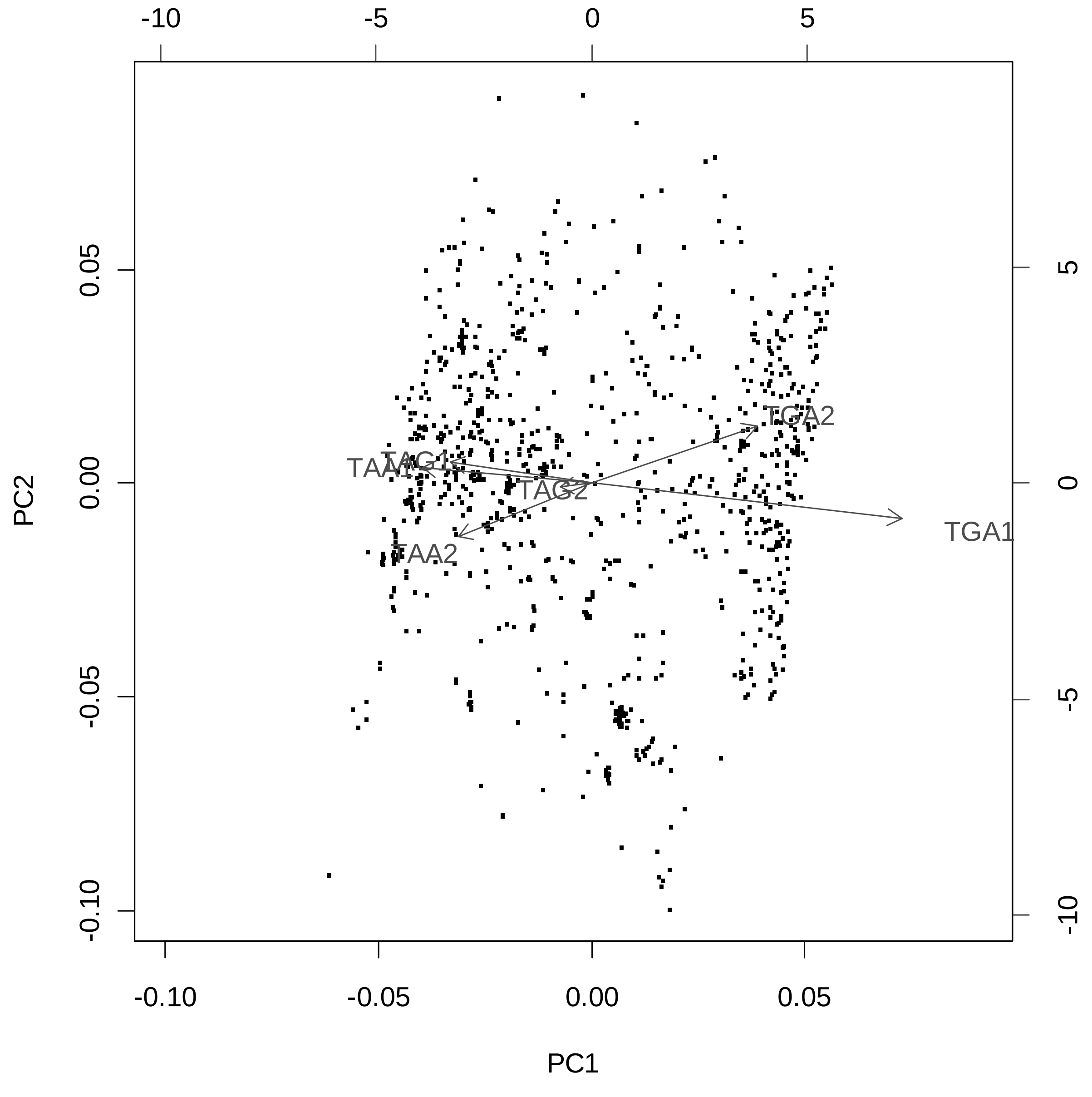

Suppl. figure **S3**

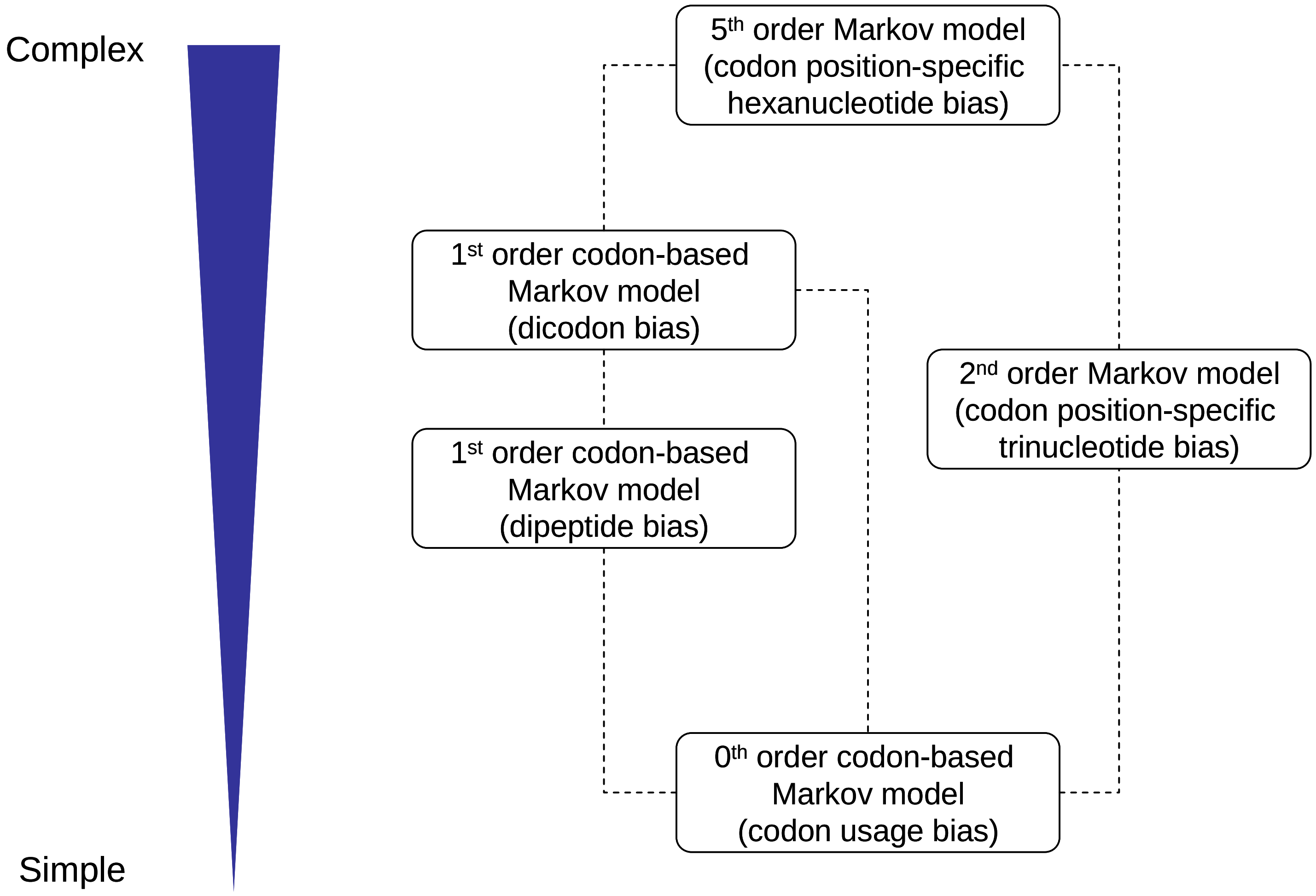

Supplement: Additional file 1 — Supplementary Table S1 and Supplementary Figures S1 to S3. Table S1, list of analyzed genomes; Figure S1, scree plot of the principal component analysis (PCA) on the relative codon frequencies of off-frame stop codons (OSC); Figure S2, biplot of the PCA results; Figure S3, relationship of the Markov models used. [file 1471-2164-11-491-S1.PDF]
